# *In situ* tumor-triggered subcellular precise delivery of multi-drugs for enhanced chemo-photothermal-starvation combination antitumor therapy

**DOI:** 10.7150/thno.52000

**Published:** 2020-10-27

**Authors:** Xinglu Jiang, Xiaobo Fan, Rui Zhang, Wei Xu, Hailu Wu, Fengfeng Zhao, Han Xiao, Chen Zhang, Chenggui Zhao, Guoqiu Wu

**Affiliations:** 1 Medical School of Southeast University, Nanjing 210009, People's Republic of China.; 2 Center of Clinical Laboratory Medicine, Zhongda Hospital, Southeast University, Nanjing 210009, People's Republic of China.; 3 Jiangsu Provincial Key Laboratory of Critical Care Medicine, Southeast University, Nanjing 210009, People's Republic of China.

**Keywords:** drug combination therapy, self-assembling peptide, triggered drug release, subcellular drug delivery, size and charge switches.

## Abstract

**Rationale:** Drug combination therapy for cancer treatment exerts a more potent antitumor effect. The targeted delivery and release of multiple drugs in a patient's body thus presents a more effective treatment approach, warranting further research. **Methods:** Two antitumor drugs (ICG: indocyanine green and THP: pirarubicin) were successfully screened to sequentially trigger self-assembling peptides (P60) to produce bacteria-sized particles (500-1000 nm, P60-ICG-THP). First, after mixing equal amount of P60 and ICG, trace amount of water (the mass ratio between P60 and water: 100:1) was used to trigger their assembly into P60-ICG. Subsequently, the assembly of P60-ICG and THP was further triggered by ultrasound treatment to produce P60-ICG-THP. **Results:** P60-ICG-THP constituted a cluster of several nanoparticles (50-100 nm) and possessed a negative charge. Owing to its size and charge characteristics, P60-ICG-THP could remain outside the cell membrane, avoiding the phagocytic clearance of blood and normal tissue cells *in vivo*. However, after localizing in the tumor, the size and charge switches of P60-ICG-THP, rapidly triggered by the low pH of the tumor microenvironment, caused P60-ICG-THP to segregate into two parts: (i) positively charged nanoparticles with a size of approximately 50 nm, and (ii) negatively charged particles of an uneven size. The former, mainly carrying THP (chemotherapeutic agent), could immediately cross the cell membrane and deliver pirarubicin into the nucleus of tumor cells. The latter, carrying ICG (used for photothermal therapy), could also enter the cell via the endocytosis pathway or accumulate in tumor blood vessels to selectively block the supply of nutrients and oxygen (cancer starvation). Both these particles could avoid the rapid excretion of ICG in the liver and were conducive to accumulation in the tumor tissue for photothermal therapy. **Conclusion:** Our drug delivery system not only achieves the precise subcellular delivery of two anticancer drugs due to their size and charge switches in the tumor site, but also provides a new strategy to combine chemotherapy, photothermal therapy, and cancer starvation therapy for the development of a highly efficient antitumor therapeutic regimen.

## Introduction

Targeted drug delivery (TDD) can provide a safe therapeutic strategy to selectively deliver drug molecules and therapeutic genes to diseased regions 1-4. Over the past years, a large number of smart nanomaterials have been developed to control drug release through changes in their internal environment (pH, enzyme concentrations, and oxidation) and external operators remotely controlling these conditions (magnetic force, temperature, and light intensity) 5-8. There have been rapid advancements in the development of these controlled drug delivery systems for improving drug safety and efficacy; however, most of these systems can only target a single type of cell or tissue to deliver a single drug 6, 9. The therapeutic effects of single drugs for cancer are limited; hence, drug combination therapies, such as combined chemotherapy, chemo-photodynamic therapy, and chemo-immunotherapy, have recently attracted more attention 10-13. However, the intracellular sites of action of different antitumor drugs are different. For example, pirarubicin (THP), cisplatin, and adriamycin act on DNA in the cell nucleus, while paclitaxel prevents the depolymerization of microtubulin filaments in the cytoplasm; furthermore, another antitumor drug, indocyanine green (ICG), is rapidly excreted by the liver. Hence, the targeted subcellular delivery of multiple antitumor drugs should improve the overall antitumor effect of the drugs, while further reducing the side effects. In fact, it is thus very difficult to achieve a simultaneous concentration of multiple drugs at the tumor site.

The cell membrane is a biological barrier, protecting cells from their surroundings, and its main function is to maintain cellular homeostasis by controlling the exchange of substances between internal and external environment of cells. Most nanomaterials for biomedical applications cannot achieve diagnostic and therapeutic efficacy until they cross the cell membrane and safely enter cells 14, 15. The physicochemical properties of nanomaterials, especially the particle size and surface charge, could play important roles in nanomaterial-cell interactions and directly affect their cellular uptake 16-18. Some studies have found that 50-150 nm is the optimum size for achieving the highest cellular uptake, since cellular uptake efficiency decreases with increasing particle size 19-21. The positively charged nanoparticles get attracted to the cell membrane and show a higher phagocytic uptake than the negatively charged ones, as the cell membrane is composed predominantly of a phospholipid bilayer and the adhering membrane proteins are usually negatively charged 17, 22, 23. Nevertheless, some researchers have demonstrated that nanoparticles with a negative charge can avoid clearance of the reticuloendothelial system (RES) in the liver to gain blood compatibility, having greater potential to deliver antitumor drugs more efficiently to the tumor sites 24, 25. Thus, specific conditions that trigger changes in the physicochemical properties of the materials should have the potential to allow precise and controlled drug release.

Self-assembled peptide-based macro-/micro-/nanomaterials could be designed and prepared via noncovalent interactions such as ionic, hydrophobic, and hydrogen bonding, and π-π stacking interactions 26-29. They exhibit well-defined nanostructures and interesting biomedical applications owing to their structural, mechanical, and functional diversity, high biocompatibility, and biodegradability 30, 31. Some stimuli, such as pH, temperature, light, and enzymes, can change the function and structure of responsive peptides, making them potential candidates for the development of biosensors, tissue engineering, drug delivery, and the so-called smart biomaterials 32-34. Human peptides have greater potential for biomedical applications as nanocarriers for drug delivery due to their biocompatibility, bioavailability, and stability in the human body.

**Scheme 1:**
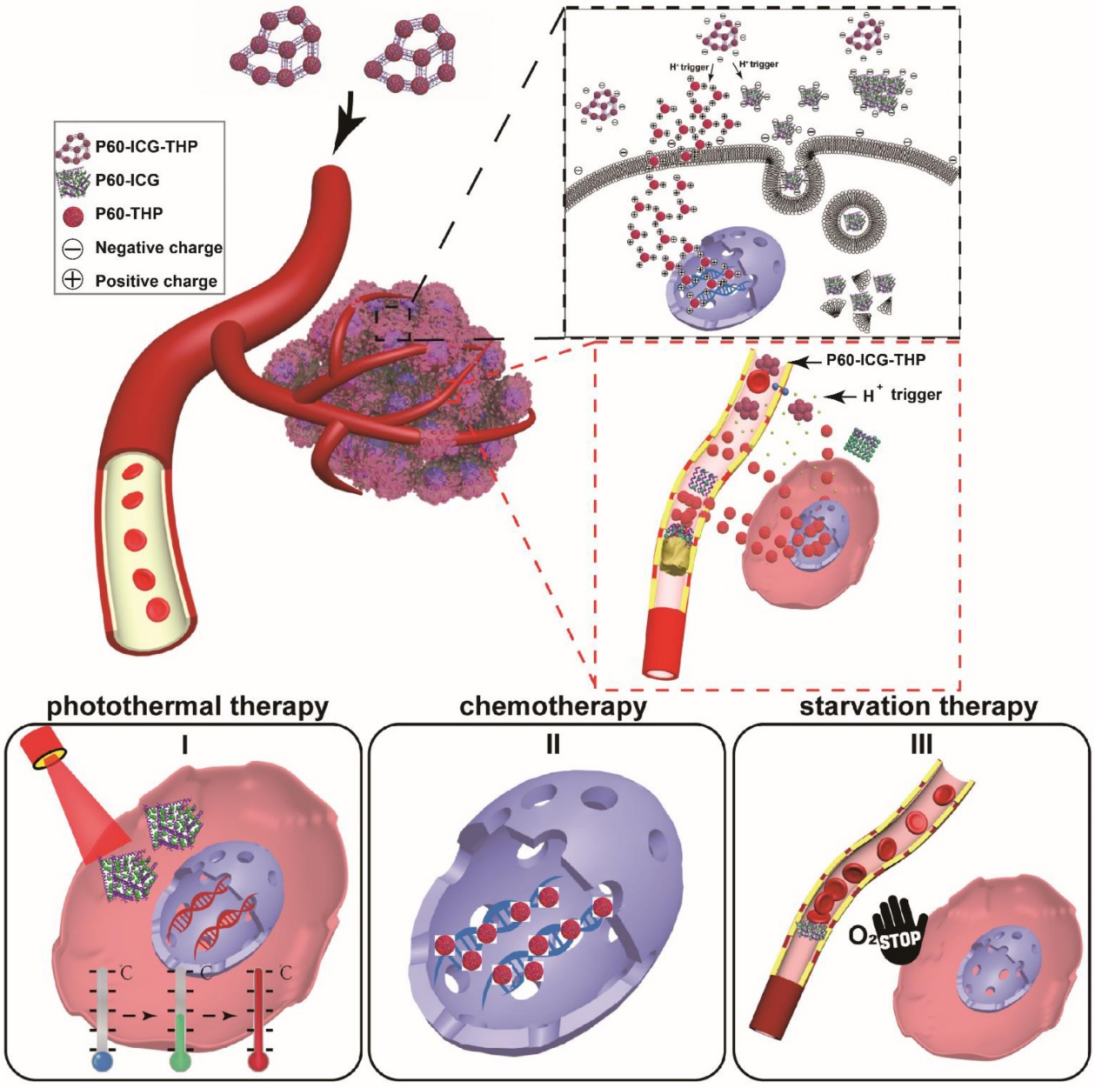
Schematic illustration of the particle size/charge switch triggered by acidic pH for precise controlled release of two antitumor drugs. Photothermal therapy, chemotherapy, and starvation therapy were combined for highly effective antitumor effect.

In this study, we screened two compounds (ICG: indocyanine green and THP: pirarubicin) from the available resource of antitumor drugs. These two compounds could be sequentially assembled by a 60-residue peptide (P60) with two α-helices to produce a bacteria-sized (500-1000 nm) negatively charged drug delivery system (P60-ICG-THP). Tumors with low pH could rapidly trigger both particle size and surface charge switches in this system. We observed that P60-ICG-THP remained outside the cell membrane at pH 7.4, while being able to precisely deliver ICG and THP into the subcellular fraction of cancer cells in response to a decrease in pH. Furthermore, the insoluble substances from the release of P60-ICG-THP in the blood vessels were found to selectively block tumor blood vessels and initiate tumor cell death. Taken together, our drug delivery system not only provides a new strategy for targeted drug delivery, but also for a combined chemo, photothermal, and cancer starvation therapy to achieve a highly efficient antitumor effect (Scheme 1).

## Methods

### Chemicals and Reagents

All chemicals were obtained from Sigma-Aldrich (St. Louis, MO, USA) unless otherwise mentioned. All antitumor drugs were purchased from Aladdin (Shanghai, China) and are enlisted in Tables S1 and S2. All buffer solutions were prepared in ultrapure water (Millipore, Billerica, MA, USA). The manufacturers of these anticancer drugs as well as the chemical formulas of the drugs can be referred to in the Chemical Book (https://www.chemicalbook.com/).

The α-helical peptide P60, (ESLVKFQAKVEGLLQALTRKLEAVSKRLAILENTVVGKIRSLHTDALKKLAVKCEDLFMA) was synthesized using a solid-phase method by China Peptides Co. Ltd. (95% purity). Secondary structure analysis was performed using ChemBio3D Ultra 12.0. The tertiary structure was obtained using the SWISS-MODEL server (https://www.swissmodel.expasy.org/interactive) and hydrophobicity/hydrophilicity analysis was performed using ProtScale online tools (http://web.expasy.org/protscale/) based on Kyte & Doolittle.

### Screening anticancer drugs to trigger peptide self-assembly

Eleven anticancer drugs (water soluble, >1 mg/mL) were screened and classified according to the number of carbon atoms and mode of water solubility. Seventeen anticancer drugs (water insoluble) were screened and classified according to the number of carbon atoms and their antitumor mechanism. The number of each category should be greater than or equal to 2.

At amounts greater than 0.5 mg, water-soluble drugs were fully blended with the same amount of P60 using a vortex oscillator and gathered via rapid centrifugation. Next, 5 μL of water was added to trigger their self-assembly. When the reaction was complete, 1 mL of water was used to sufficiently dissolve the particles. The sediment collected by centrifugation was weighed to determine the assembly of nanoparticles. After collecting the sediment, 0.5 mg of water-insoluble drugs were successively used to trigger them to form stable particles under ultrasound treatment. Their mean diameters were measured using a Zetasizer Nano ZS system (Malvern Instruments Ltd, Worcestershire, UK) at room temperature.

### Characterization of P60-ICG-THP

Particle size distribution and zeta potential were measured using the Zetasizer Nano ZS system (Malvern Instruments Ltd) at room temperature. The morphology and particle size of P60-ICG-THP were observed using transmission electron microscopy (TEM) on a JEM 2100 (JEOL, Japan) and via scanning electron microscopy (SEM) on a Zeiss Ultra Plus (Zeiss, Germany). The chemical components of P60-ICG-THP were evaluated and traced by confocal laser scanning microscopy (CLSM, FV1000, Olympus, Japan). PBS (200 μL), free ICG (200 μL, containing 100 μg/mL ICG), P60-ICG (200 μL, containing 100 μg/mL ICG), P60-ICG-THP at pH 7.4 (200 μL, containing 100 μg/mL ICG), and P60-ICG-THP at pH 6.5 (200 μL, containing 100 μg/mL ICG) were added to 96-well plates and irradiated by an 808-nm laser at 1 W/cm^2^ for 4 min. Region maximum temperatures and infrared thermographic maps were acquired using an infrared thermal imaging camera (SC620, FLIR, Switzerland).

### Mechanisms of Drug Release from P60-ICG-THP

The pH of the solution containing P60-ICG-THP *in vitro* was decreased from 7.4 to acidic pH levels. TEM (JEM 2100, JEOL) and SEM (Zeiss Ultra Plus, Zeiss) were performed to observe the morphological changes of the particles. The Zetasizer Nano ZS system (Malvern Instruments Ltd) was used to measure the change in the particle size, and fluorescence spectroscopy (FluoroMax-4, HORIBA Jobin Yvon, France) was applied to detect the variation in emission intensity, with emission at 587 nm and excitation at 480 nm to monitor TPH, and emission at 815 nm and excitation at 780 nm to monitor ICG. Gel electrophoresis and the Zetasizer Nano ZS system were used to distinguish the charges of the different particles after pH triggering and drug loading.

The *in vitro* release kinetics of pirarubicin from P60-ICG-THP were evaluated using a dialysis method. P60-ICG-THP was dissolved in three different buffer solutions: (i) saline at pH 7.4, (ii) saline at pH 6.5, and (iii) saline at pH 5.5. They were then placed in a dialysis bag (MWCO, 5500 Da) and placed in a beaker containing 30 mL of saline of the desired pH with continuous stirring at room temperature. The released pirarubicin was measured by fluorescence spectroscopy (excitation at 494 nm, emission at 587 nm).

### Cell culture

MDA-MB-231 (human breast cancer cells) cells were purchased from the National Laboratory Cell Resource (China) and were grown in Dulbecco's Modified Eagle's Medium-high glucose (GIBCO, USA) supplemented with 10% fetal bovine serum and 1% penicillin-streptomycin in a humidified incubator with 5% CO_2_ at 37°C.

### Cellular uptake of P60-ICG-THP

The cellular uptake of P60-ICG-THP was tracked by confocal laser scanning microscopy (FV1000, Olympus, Japan) and fluorescence microscopy (XSP-BM13C, Shanghai optical, China). Briefly, 5 × 10^5^ MDA-MB-231 cells were seeded on a 35-mm glass-based dish for 24 h and fresh medium containing P60-ICG-THP (20 μg/mL THP) was used to replace the spent medium. After 1 min, HCl was used to adjust the pH value. When the pH reached 6.5, the video of the emission intensity of THP was captured by fluorescence microscopy.

To distinguish the different effects on the cellular uptake of P60-ICG-THP caused by the acidic pH in the same glass-based dish, P60-ICG-THP followed by a small amount of the culture medium (pH = 6.5, 100 μL) was added to cover the surface of MDA-MB-231 cells, which was gradually covered by surface tension. Ultimately, a boundary between the solutions of pH values of 6.5 and 7.4 was formed. A fluorescence microscope was used to acquire images of the emission intensity of THP. The flow diagram of this method is presented in Figure S6.

After a 2-h incubation with free ICG (20 μg/mL), free THP (20 μg/mL), P60-ICG (20 μg/mL), and P60-ICG-THP (ICG: 20 μg/mL; THP: 20 μg/mL) in a culture medium of pH 7.4 or 6.5, the dishes were washed three times with PBS and the cell nuclei of MDA-MB-231 were stained with DAPI. DAPI, THP, and ICG were excited at 405 nm, 561 nm, and 633 nm, respectively. Their emission intensities were observed by confocal laser scanning microscopy (FV1000, Olympus).

### Cytotoxicity assay

Cell viability was evaluated using a Cell Counting Kit-8 system (CCK-8), according to the manufacturer's instructions (Dojindo Laboratory, Japan). Cells were seeded in 96-well plates at a density of 1 × 10^5^ cells/well. After growing overnight, they were incubated with different concentrations of free THP, free ICG, and P60-ICG-THP for 2 h in culture media of different pH levels. After 2 h, the cells in the pH 6.5 culture medium were irradiated by an 808-nm laser and the spent medium was replaced with fresh medium. The cells were cultured for an additional 24 h before conducting cell viability assay using CCK-8.

### Cancer starvation therapy model *in vitro* and *in vivo*

Cotton, soaked in a pH 5.5 buffer for 5 min, was inserted into glass capillary tubes (inner diameter = 0.3 mm). A 100 μL solution containing P60-ICG-THP (ICG: 50 μg/mL) was passed through the cotton for 30 min using a micro-injection pump. Confocal laser scanning microscopy (FV1000, Olympus, Japan) was used to track the emission intensity of ICG and THP, after excitation at 561 nm and 633 nm, respectively.

Six-week-old female athymic nude mice were inoculated subcutaneously in the left hip with approximately 1 × 10^7^ MDA-MB-231 cells to establish a xenograft model. When the long diameter of the tumor reached approximately 1 cm, P60-ICG-THP with a concentration of 20 mg/kg pirarubicin was injected into the mice through the caudal vein every day. After 24 h, the mice were euthanized and the tissues and tumors were collected for the preparation of tissue sections and immunofluorescence assays. The cell nuclei were stained with DAPI, and vascular endothelial cells were stained with FITC-CD31. The emission intensities of DAPI, FITC, THP, and ICG were obtained by confocal laser scanning microscopy, excited at 405 nm, 488 nm, 561 nm, and 633 nm, respectively.

After the mice xenograft models were euthanized, the fresh tumors and liver tissues were lysed for ATP and H_2_O_2_ content determination. The ATP content in the supernatant was measured using an ATP assay kit (Beyotime Biotechnology, China). The HIF-α levels in the supernatant was measured using a HIF-1α ELISA Kit (Beyotime Biotechnology, China). The total protein concentration was measured with a Bicinchoninic Acid (BCA) Protein Quantitative Analysis Kit (Beyotime).

### *In vivo* antitumor activity and tumor-targeting biodistribution study

After approximately 3 days, the tumor size of the xenograft model reached approximately 50 mm^3^. The mice were then randomly divided into five groups, and injected with PBS, P51, free ICG, free THP, P60-ICG-THP, or P60-ICG-THP at a concentration of 20 mg/kg THP or ICG, through the caudal vein every alternate day. Tumor lengths, widths, and mouse weights were recorded daily (tumor volume = length × width × width/2). After 21 days of treatment, the mice were euthanized. Tissues and tumors (Figure S13) were collected in a 4% formaldehyde solution and embedded in paraffin for hematoxylin and eosin (H&E) staining. Blood was obtained from the eyeballs of mice and used for biochemical detection and blood cell analysis. Institutional guidelines for the proper and humane use of animals were followed.

IVIS Spectrum *In vivo* Imaging System and *in vivo* thermal imaging were used to study the biodistribution of P60-ICG-THP. The xenograft model (long diameter = 1 cm) was injected with P60-ICG-THP (ICG: 50 mg/kg) via the caudal vein. At three time points (0.5, 12, and 25 h), the fluorescence images of the mice (the emission intensity of THP) and thermal images were recorded. Before thermal imaging, the tumors were exposed to an 808-nm laser at a power density of 1 W/cm^2^ for 5 min.

### Statistical analysis

All data are presented as the mean and standard errors of the mean from at least three independent experiments. Student's t-test or one-way ANOVA was used for statistical analysis, and p < 0.05 was considered statistically significant.

## Results and Discussion

### Two anticancer drugs screened to trigger peptide self-assembly

To avoid an unfavorable immune response, P60, which contains a sequence of 60 amino acids from two human proteins, was first introduced to load drugs. It includes two α-helix sequences from human matrilin-1 (ESLVKFQAKVEGLLQALTRKLEAVSKRLAILENT) 35, 36 and pseudopodium-enriched atypical kinase 1 (VVGKIRSLHTDALKKLAVKCEDLFMA) 37. The characteristics of the three peptides (P60, and the two α-helix sequences) are shown in Table S1 and Figure S1. In solution at pH 7, P60 possesses a net positive charge of 4.1 and the positively charged P60 can enhance the drug-loading rate of negatively charged drugs.

**Figure 1:**
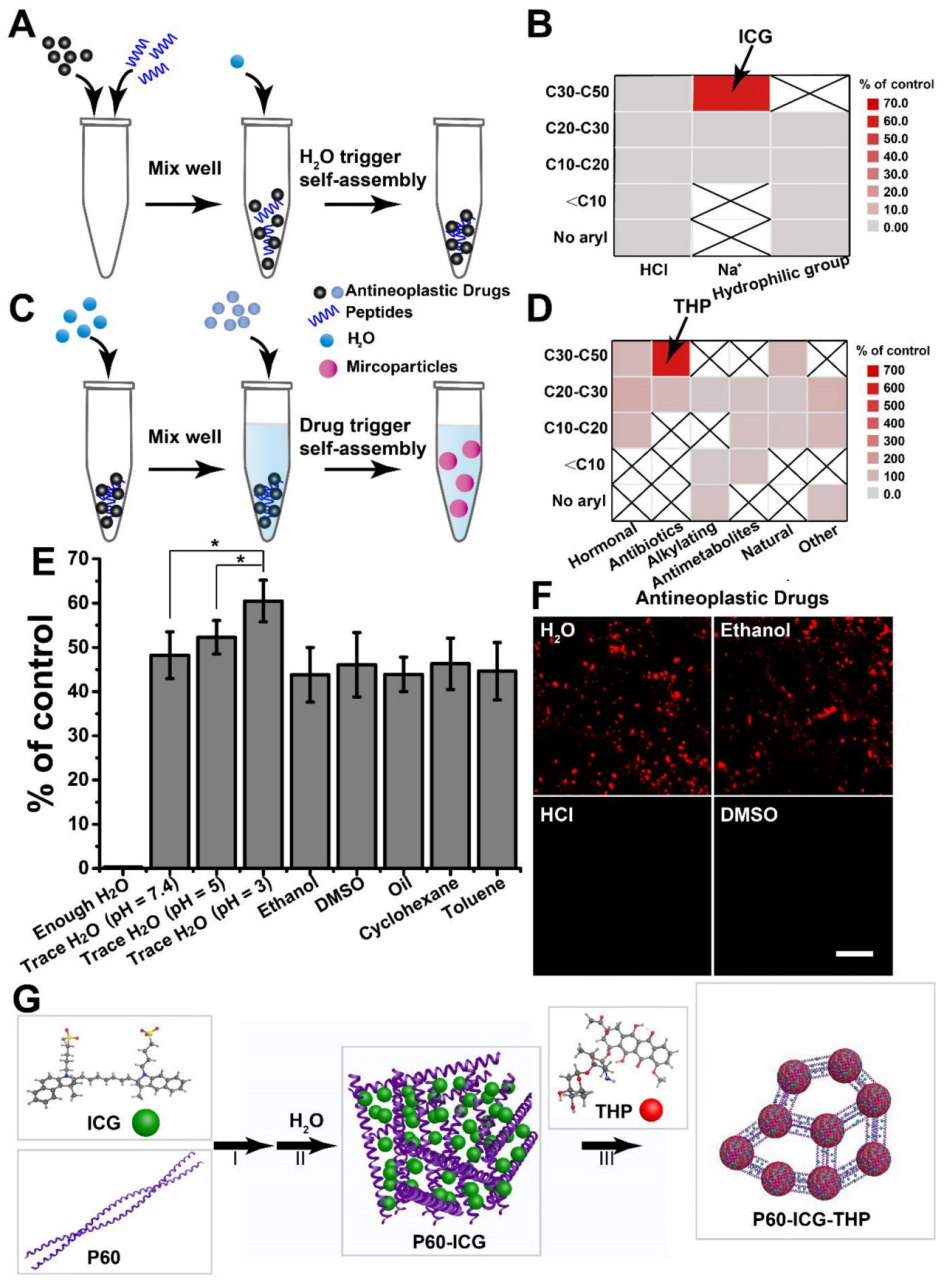
** Screening of the antitumor drugs forming large-sized and negatively charged particles. (A)** and **(C)** Illustration of peptide self-assembly triggered by H_2_O and drug, respectively.** (B)** and **(D)** Results of the screening. C10, C20, C30, and C50: number of carbon atoms in the antitumor drug molecules. **(E)** Different solvents replace H_2_O to trigger peptide self-assembly, *p < 0.05. **(F)** Different solvents are used to mix THP and P60-ICG. Scale bar = 20 μm. **(G)** The schematic representation of the synthesis procedure of P60-ICG-THP.

Most anticancer drugs containing a lipophilic group, especially the aryl group, are water insoluble. To increase their water solubility, some of them can be prepared as hydrochlorides (HCl) and ionic compounds (Na^+^), or be modified by a hydrophilic group. According to our previous study 5, 38, two different methods to successively load water-soluble and water-insoluble anticancer drugs were designed; their flow-diagrams are presented in Figure 1A and Figure 1C, respectively. Based on the number of carbon atoms and the mode of water solubility, 11 water-soluble anticancer drugs (solubility ≥ 1 mg/mL, Table S2) were selected to assemble with P60. The sediments were gathered and weighed after self-assembly, and the results are shown in Figure 1B. Sedimentation was observed only with the indocyanine green (ICG) and P60 group, indicating that traces of water can trigger their self-assembly. Then, the sediment containing P60-ICG was triggered by 17 water-insoluble anticancer drugs (Table S3) using ultrasound treatment. The comparative particle changes and the results for each tested water-insoluble drug are shown in Figure 1D. We found that stable particles were obtained only with THP, whereas the particle size did not change in the groups of other water-insoluble anticancer drugs. The above results showed that THP and ICG could trigger P60 self-assembly.

To better understand the mechanism of the two anticancer drugs loaded by P60 self-assembly, the hydrosolvent was replaced with another solvent. As shown in Figure 1E, we found that trace amounts of polar and nonpolar solvents could also trigger the assembly of P60 and ICG, and lower pH appeared to be more conducive for achieving the same. When enough water was added, ICG and P60 were completely dissolved and assembly did not occur. The results illustrated that electrostatic interactions were the main binding forces between P60 and ICG. When the solubility of THP was increased through the use of hydro-chlorination and a polar solvent, the particles were not obtained (Figure 1F). This illustrated that the hydrophobic effect of THP played a crucial role in the formation of these particles (P60-ICG-THP). The schematic diagram of the self-assembly of P60 with ICG and THP is presented in Figure 1G.

### Characterization of P60-ICG-THP

**Figure 2:**
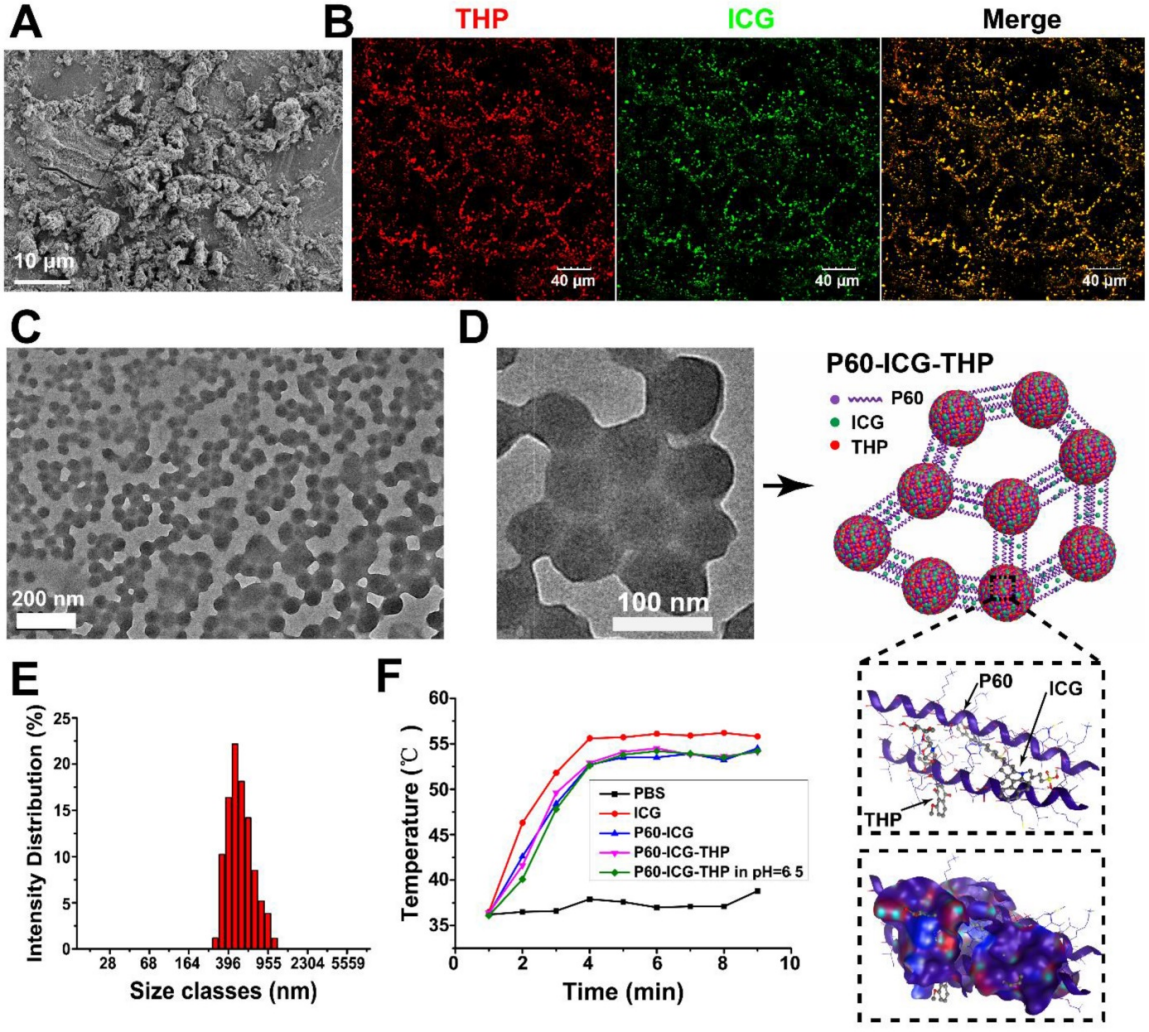
** Characterization of P60-ICG-THP. (A)** SEM image of P60-ICG. **(B)** Confocal fluorescence images for the tracing of ICG (green) and THP (red) in P60-ICG-THP. **(C)** and **(D)** TEM images of P60-ICG-THP and an illustration of its structure. **(E)** Particle diameter of P60-ICG-THP. **(F)** Maximum infrared temperature profiles of PBS, ICG, P60-ICG, P60-ICG-THP, and P60-ICG-THP at pH = 6.5 under continuous 808-nm laser irradiation.

After low-speed centrifugation of the reaction solution containing P60 and ICG, a green sediment appeared at the bottom of the centrifuge tubes (see Figure S2A), indicating that P60 and ICG were successfully assembled. Many non-homogeneous particles were observed in the SEM image of P60-ICG (Figure 2A). We first used confocal microscopy to track the emission intensity of THP and ICG to verify whether the two drugs were assembled in P60-ICG-THP (Figure 2B). The diameter of P60-ICG-THP was approximately 500-1000 nm, as observed in the confocal images. After the confocal images were merged, the red and green fluorescence signals turned yellow because of the co-localization of ICG and THP, confirming that the two drugs were bound together by P60. TEM images of P60-ICG-THP (Figure 2C and Figure 2D) showed spherical nanostructures with diameters of approximately 50-100 nm distributed in full view, and several nanospheres were clustered by linking with each other to form particles with a size of 300-1000 nm, consistent with the results of confocal microscopy. Based on the zoomed-in TEM image, we could draw the structure diagram of P60-ICG-THP (Figure 2D). The diameter of P60-ICG-THP was measured by dynamic light scattering (Figure 2E) to be 513 ± 210 nm, consistent with the results of confocal microscopy and TEM, and it was similar to the size of bacteria such as *Escherichia coli* and* Staphylococcus aureus*. The fluorescence intensity of ICG and THP from the delivery system (P6-ICG-THP) was measured at different time points to assess its stability in the serum. The results revealed no noticeable change in the fluorescence intensity of ICG and THP as shown in Figure S4. The temperature variations of phosphate-buffered saline (PBS), free ICG, P60-ICG, P60-ICG-THP, and P60-ICG-THP in buffer (pH 6.5) 5 min after 808-nm laser irradiation at 1W/cm^2^ are shown in Figure S5 (infrared thermographic maps) and Figure 2F (maximum temperature profiles). We found that acidic environments had no effect on the photothermal efficiency of ICG in P60-ICG-THP. The results confirmed that photothermal therapy using P60-ICG-THP could be applied in acidic tumor microenvironments.

### Acidic pH triggers particle size and charge switches

**Figure 3:**
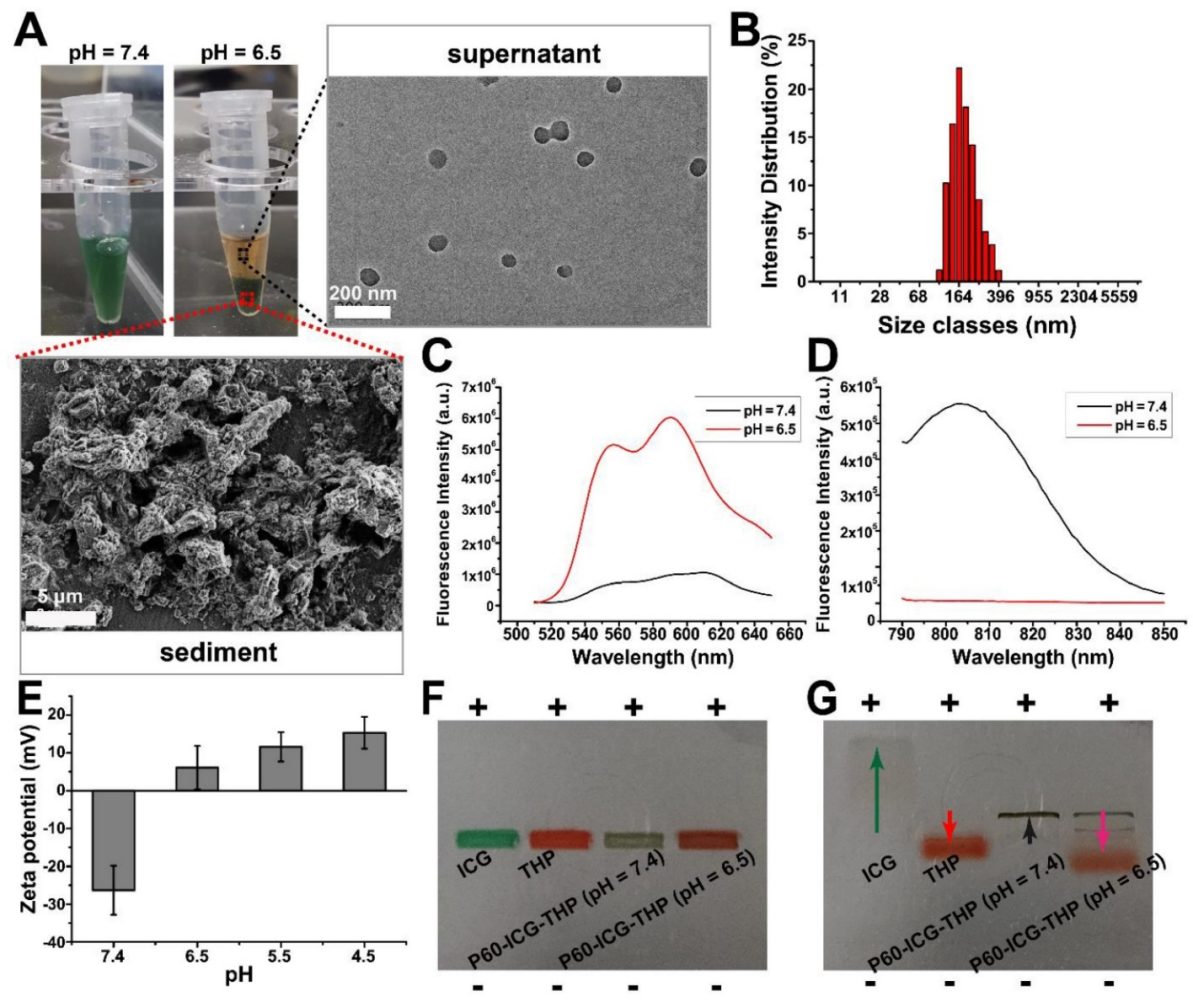
** Acidic pH triggers the particle size and charge switch of P60-ICG-THP. (A)** The photographs of P60-ICG-THP at different pH values. Magnified regions: TEM image of the supernatant obtained after acid treatment and SEM image of the sediment obtained after acid treatment. **(B)** Particle diameter of the supernatant at pH 6.5.** (C)** and **(D)** The emission intensity of THP and ICG in P60-ICG-THP at different pH values. **(F)** Zeta potential of P60-ICG-THP at different pH levels. **(F)** Before and **(G)** After electrophoresis, +: positive pole, -: negative pole.

The tumor microenvironment is often acidic, and acidic metabolites such as lactic acid produced by anaerobic glycolysis during hypoxia, appear to be the main underlying cause 39, 40. Therefore, the acidic pH of tumors is often designated as a target stimulus trigger for the controlled release of antitumor drugs 9, 41. When the pH of the solution containing P60-ICG-THP was buffered close to 6.5, green sediments were separated and precipitated at the bottom of the centrifuge tube after a few minutes, and the supernatant became red (pH 6.5, Figure 3A). The TEM image of the supernatant (the left bottom portion of Figure 3A) showed that nanospheres with diameters of approximately 100-150 nm were released from P60-ICG-THP and were dominated by a red color. The average diameter of the particles in the supernatant (Figure 3B), as measured by dynamic light scattering, became smaller (150 ± 38 nm), which was conducive to cell membrane crossing. The SEM image of the green sediment (upper right portion of Figure 3A) was similar to that of P60-ICG, and they were also non-homogeneous particles. The *in vitro* release kinetics of pirarubicin from P60-ICG-THP was shown in Figure S2C, which was in correspondence with the above results.

To further analyze their composition, the emission intensities of THP and ICG in P60-ICG-THP were measured by fluorescence spectrophotometry at pH 7.4, or in buffer at pH 6.5 (Figure 3C-3D). From pH 7.4 to 6.5, the emission intensity of P60-ICG-THP was observed as follows: the emission intensity of THP increased by ten-fold, while that of ICG was reduced by one hundred-fold. Fluorescence resonance energy transfer (FRET) could be responsible for the increase in the emission intensity of THP because it was released from P60-ICG-THP in the buffer at pH 6.5, and the fluorescence quenching of ICG resulted from its own aggregation. These results from fluorescence spectrophotometry also illustrated that a low pH could trigger P60-ICG-THP to release ICG and THP. The nanospheres in the supernatant mainly carried THP, while most of the ICG was caught in the sediment.

The surface charges of P60-ICG-THP were measured at different pH values, and the results are shown in Figure 3E. At pH 7.4, the zeta potential of P60-ICG-THP was negative (-26.3 ± 6.5), while it became positive as pH values decreased. Gel electrophoresis was used to further evaluate the charge properties of ICG, THP, and P60-ICG-THP. The gel images before and after electrophoresis are shown in Figure 3F-3G, respectively. The ICG (negatively charged molecule) and P60-ICG-THP in a pH 7.4 buffer moved to the positive pole while THP (positively charged molecule) moved to the negative pole. For the P60-ICG-THP in the pH 6.5 buffer, three electrophoretic bands were obtained. Based on the results of fluorescence spectroscopy (Figure 3C-3D), by knowing their location in the gel and their color, we could identify the components of the three bands. The top band was assumed to be P60-ICG (negative charge), since its diameter is relatively large. The following two bands should be P60-THP (positive charge). The movement speed of large particles was slow, so they should be in the middle band. The zeta potential results were consistent with those of the gel electrophoresis.

**Figure 4:**
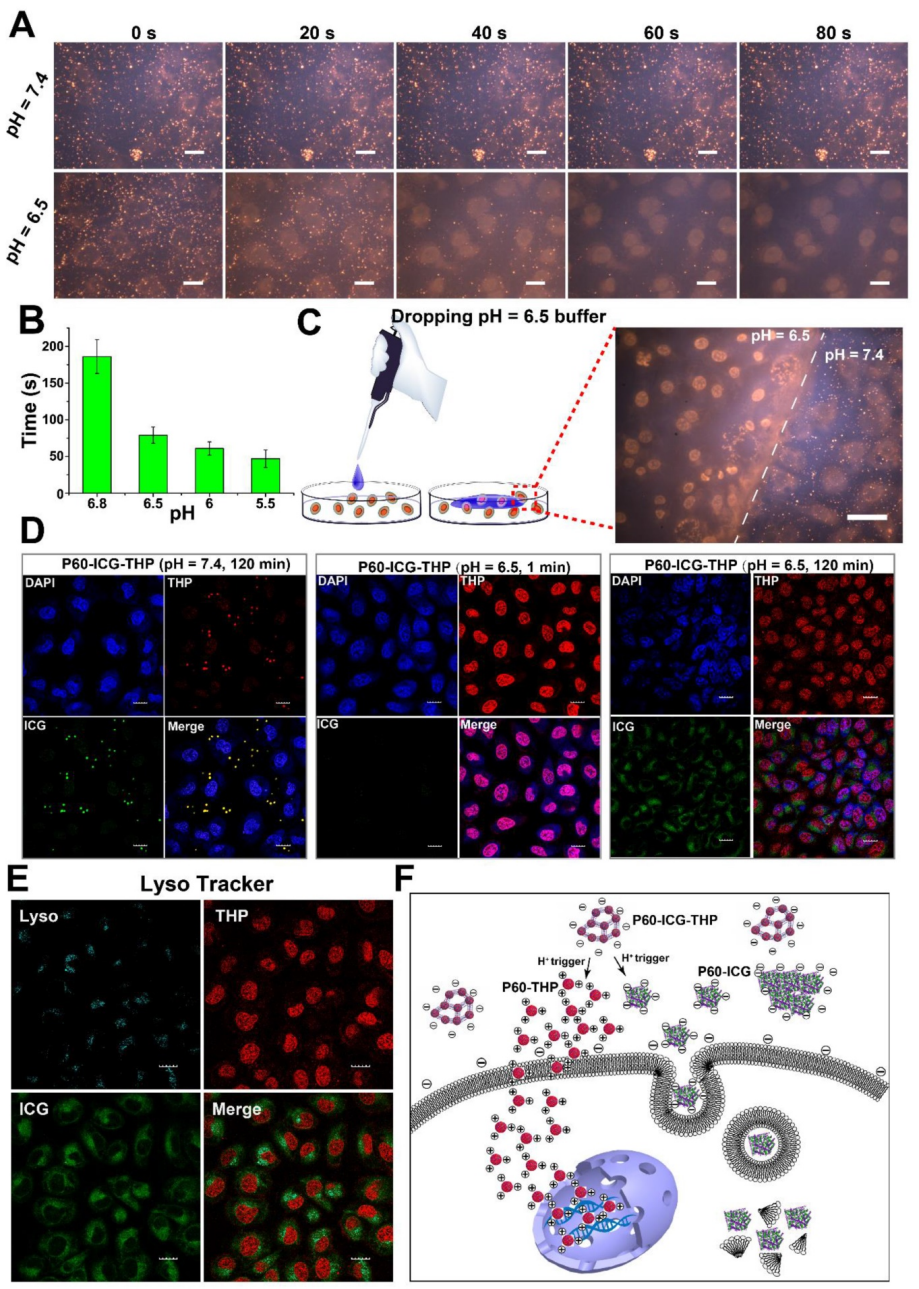
** (A)** THP fluorescence images of MDA-MB-231 cells incubated with P60-ICG-THP at pH 6.5 and 7.4 for different time points (the images were collected from the video provided in the supporting material). Scale bar = 20 μm. **(B)** The minimum time of fluorescence disappearance of P60-ICG-THP incubated with MDA-MB-231 at different pH values (n = 3). **(C)** THP fluorescence images of MDA-MB-231 incubated with P60-ICG-THP after treating with a drop of pH = 6.5 buffer in the same culture dish, scale bar = 20 μm. **(D)** The confocal microscope images of MDA-MB-231 incubated with P60-ICG-THP at different time points at different pH levels. Cells were stained with DAPI (blue), THP (red), and ICG (green) excited at 405 nm, 561 nm and 633 nm, respectively. Scale bar = 20 μm.** (E)** Endosome/lysosome labeling by Lyso Tracker, scale bar = 20 μm. **(F)** Schematic illustration of the cellular uptake of two drugs triggered by an acidic pH.

In summary, low pH rapidly triggers the size/charge switch of P60-ICG-THP into two particles: (i) positively charged nanoparticles with a size of approximately 50-100 nm and (ii) negatively charged particles of an uneven size (Figure 4F). The former mainly carried THP, while the latter mainly loaded ICG.

### Cellular uptake triggered by acidic pH and the subcellular precise delivery of ICG and THP

To validate the feasibility of using P60-ICG-THP for acidic pH-triggered cellular uptake *in vitro*, human breast cancer cells (MDA-MB-231) were incubated with P60-ICG-THP in culture medium at pH 7.4 and 6.5. The emission intensity of THP was recorded at different time points using a video capture system. As shown in the video images in Figure 4A, the fluorescence of MDA-MB-231 cells treated with P60-ICG-THP in culture medium at pH 7.4 showed no obvious change from 0 to 80 s. This suggested that the large size and negative charge of the drug delivery system could not cross the cell membrane in a pH 7.4 environment. Furthermore, this was beneficial to avoid phagocytic clearance of blood and normal tissue cells in the body circulation.

In a pH 6.5 environment, the red fluorescent particles (P60-ICG-THP) gradually disappeared and the intensity of red fluorescence in the cell nuclei became stronger. The clearance time of all red fluorescent particles at different pH values was calculated (Figure 4B). With decreasing pH, the time needed for P60-ICG-THP to disappear continuously decreased. In a pH 6.8 culture medium, it took about 3 min, while in a pH 5.5 culture medium, it took less than a minute. These data illustrated that P60-ICG-THP was ultrasensitive under an acidic pH. To further assess its specific response to an acidic pH, culture medium of pH 6.5 was added dropwise to the surface of MDA-MB-231 cells that were incubated with P60-ICG-THP (the flow diagram of this method is shown on the left portion of Figure 4C and Figure S6). In Figure 4C, a dividing line (white dashed line) distinguishes different areas based on pH value, and the THP released from our delivery system crossed the cells in the culture medium at pH 6.5, reaching the nucleus. These results implied that an acidic pH could rapidly trigger the cellular uptake of the tested drugs, achieving precise, pH-responsive drug release.

Next, the subcellular distributions of THP and ICG after co-incubation with P60-ICG-THP at pH 7.4 for 120 min, at pH 6.5 for 1 min, and at pH 6.5 for 120 min were observed by confocal microscopy (Figure 4D). From the results, we concluded that both ICG and THP could remain outside the cell at pH 7.4, but acidic pH could trigger THP to immediately cross the cell membrane and enter the nucleus, while ICG was mainly located in the cytoplasm even after approximately 120 min. Through lysosomal labeling (Figure 4E), the emission intensity of ICG was observed in the lysosomes, while the emission intensity of THP was not. This means that the mechanism by which they enter the cell was different, and that THP and ICG enter the cell via direct translocation and the endocytic pathway, respectively. Ultrasensitive pH-triggered differences in sizes and surface charge switches could be the main underlying causes.

In an acidic environment (Figure 4F), H^+^ can immediately trigger the large-sized and negatively charged P60-ICG-THP to divide into two smaller particles: (i) approximately 50-nm-sized nanoparticles with a positive charge and (ii) uneven-sized particles with a negative charge. The positively charged nanoparticles, mainly loaded with THP, can immediately cross the cell membrane and deliver pirarubicin into the nucleus. The negatively charged particles carrying ICG can enter the cell cytoplasm via the endocytosis pathway.

### Chemo-photothermal combination therapy *in vitro*

**Figure 5:**
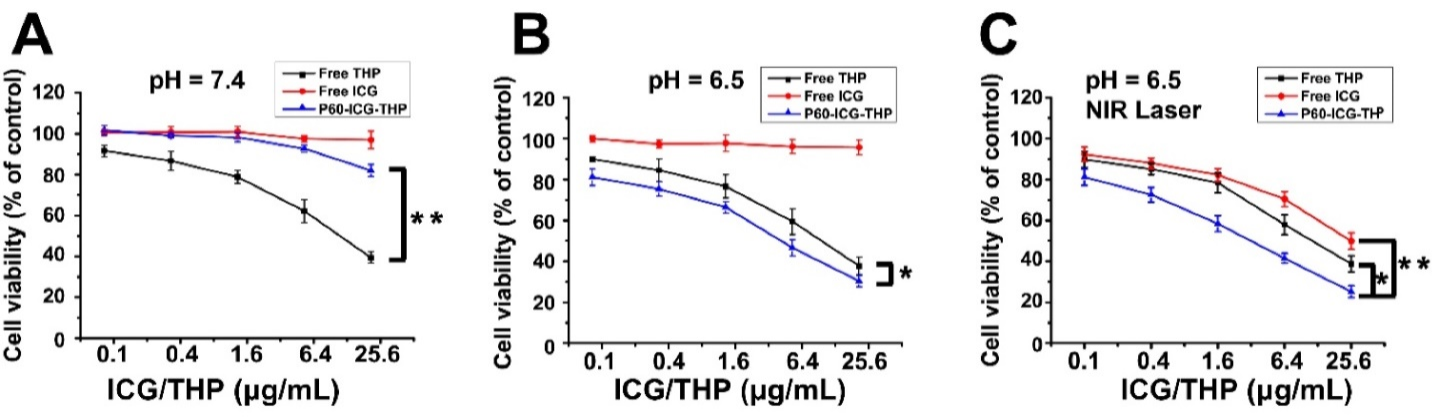
** (A-C)** The viability of MDA-MB-231 cells incubated with different concentrations of free THP, free ICG, and P60-ICG-THP at pH 7.4 or 6.5, with or without NIR laser irradiation. *p < 0.01, **p < 0.001.

To evaluate the cytotoxic effect of chemo-photothermal treatment *in vitro*, MDA-MB-231 cells were incubated in culture media at pH 7.4 and 6.5, containing the same concentrations of free ICG, free THP, and P60-ICG-THP with or without near-infrared (NIR) laser irradiation (Figure 5A-5C). At pH 7.4, P60-ICG-THP and free ICG did not inhibit cancer cell growth (Figure 5A), suggesting their biocompatibility, whereas a greater number of cancer cells were killed with increasing amounts of free THP. In an acidic environment, the cytotoxic effect was markedly enhanced, and was greater than that of free THP alone. Cell viability decreased from 82.01% to 30.33% after treatment with 25.6 μg/mL THP (p < 0.01, Figure 5B). These results imply that our drug delivery system based on acidic pH-triggered release could play an important role in targeted therapy. To investigate photothermal therapeutic efficacy, MDA-MB-231 cells treated with free ICG, free THP, and free P60-ICG-TH were exposed to NIR laser irradiation for 5 min after H^+^ trigger. Free ICG at 25.6 μg/mL killed 50.13% of cancer cells after NIR laser irradiation. However, the chemo-photothermal combination therapy of P60-ICG-THP evidently resulted in significantly lower cell viability than chemo- or photothermal treatment alone (p < 0.01 for all, free THP vs. free ICG vs P60-ICG-THP: 38.73% vs. 49.87% vs. 25.11%, Figure 5C).

### Cancer starvation therapy through selective occlusion of tumor blood vessels

To undergo metabolism and growth, solid tumor cells and tissues require more nutrients and energy compared to their nontumor counterparts; therefore, cancer starvation therapy is a burgeoning method to suppress tumor growth by blocking nutrient supply 42-44. Selective occlusion of tumor blood vessels can be one of the methods to interrupt the supply of fundamental nutrients and oxygen to the tumor for cancer treatment 45-47. We found that acidic pH can trigger P60-ICG-THP to release insoluble P60-ICG. Here, *in vivo* simulation was designed to verify whether the insoluble P60-ICG could block the capillary tube (schematic diagram shown in Figure S8). Using a micro-injection pump, a solution containing P60-ICG-THP was slowly flowed past the acidic cotton. After 30 min, the white cotton became dark green (black arrow in the top-left photograph of Figure S9A-S9B), suggesting that P60-ICG was deposited around the acidic cotton and might block the capillary tube. Confocal microscopy was performed to track ICG and THP in the capillary tubes, and the fluorescence images are shown in Figure S9A-S9B. Compared with the control group (pH 7.4), P60-ICG-THP disappeared after passing through the acidic cotton. At the same time, the emission intensity of ICG decreased while that of THP increased. The results were consistent with the reversal of the emission intensity of P60-ICG-THP in the acidic buffer, which could provide a theoretical basis for the selective occlusion of tumor blood vessels triggered by acidic pH.

**Figure 6:**
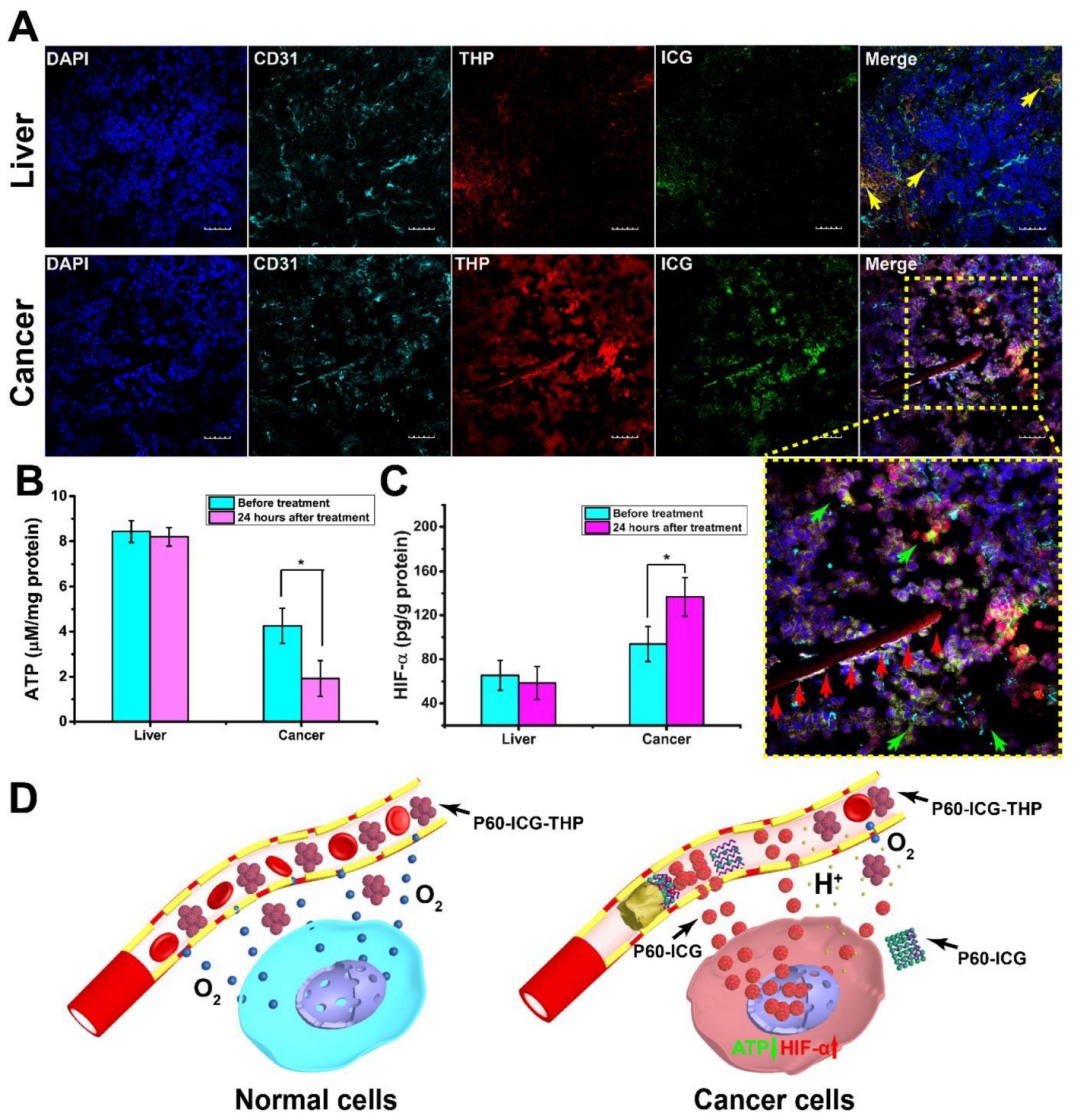
** Cancer starvation therapy *in vivo*. (A)** Confocal fluorescence images of the liver and tumor tissues from mouse xenograft models treated with P60-ICG-THP. Cell nuclei were stained with DAPI and vascular endothelial cells were stained with FITC-CD31. DAPI (blue), FITC (cyan), THP (red), and ICG (green) were excited at 405 nm, 488 nm, 561, and 633 nm, respectively. Scale bar = 20 μm. The yellow arrows show P60-ICG-THP, the green arrows show ICG released from P60-ICG-THP, and the red arrows show that P60-ICG-THP blocks the blood capillary. Lower right image: enlarged image. **(B)** ATP and** (C)** HIF-α in the liver and tumor tissues from xenograft models treated with P60-ICG-THP (n = 3, *p < 0.01).** (D)** Schematic illustration of starvation therapy of P60-ICG-THP *in vivo*.

To further demonstrate that P60-ICG-THP can selectively block tumor blood vessels after acid activation *in vivo*, mice bearing MDA-MB-231 tumors were treated with P60-ICG-THP via tail vein injection. After 24 h, the visceral organs and tumor tissues were harvested and subjected to DAPI and CD31 staining to label the nuclei and vascular endothelial cells, respectively. A strip of red emission intensity (red arrows in Figure 6A) was found to fill only the tumor blood vessels, while the green ones vanished. Furthermore, this result meant that THP and ICG accumulated more efficiently in the tumor. It was surprisingly consistent with the reversal of emission intensity in the capillary tube, demonstrating that P60-ICG-THP had the ability to selectively block tumor blood vessels. Compared with live tissues, the decreasing concentration of ATP (Figure 6B) and increasing concentration of HIF-α (Figure 6C) and H_2_O_2_ (Figure S10) in the tumor also explained that hypoxia had already occurred after the injection of P60-ICG-THP. A schematic representation of this mechanism of cancer starvation therapy is shown in Figure 6D.

### Precise controlled release of two anticancer drugs* in vivo*

The emission intensities of THP and ICG were significantly stronger in tumor tissues than in the visceral organs (Figure 6A and Figure S11), and there was no emission of THP observed in the cell nuclei of the visceral organs (Figure 6A and Figure S11), indicating that P60-ICG-THP exhibited a good effect on targeted drug delivery. The nuclei of the tumor tissues became purple after the co-localization of DAPI and THP, indicating that THP only targeted the cancer cell nuclei. The emission intensities of ICG were mainly located in the cancer cell cytoplasm, which was consistent with the results of the *in vitro* experiment. In particular, a number of yellow particles representing the co-localization of ICG and THP in the visceral organs illustrated that the P60-ICG-THP were intact particles (yellow arrows in Figure 6A and Figure S11). Because the pH of the visceral organs was approximately 7.35-7.45, P60-ICG-THP particles with a negative charge and large size could avoid phagocytic clearance in the body circulation.

**Figure 7:**
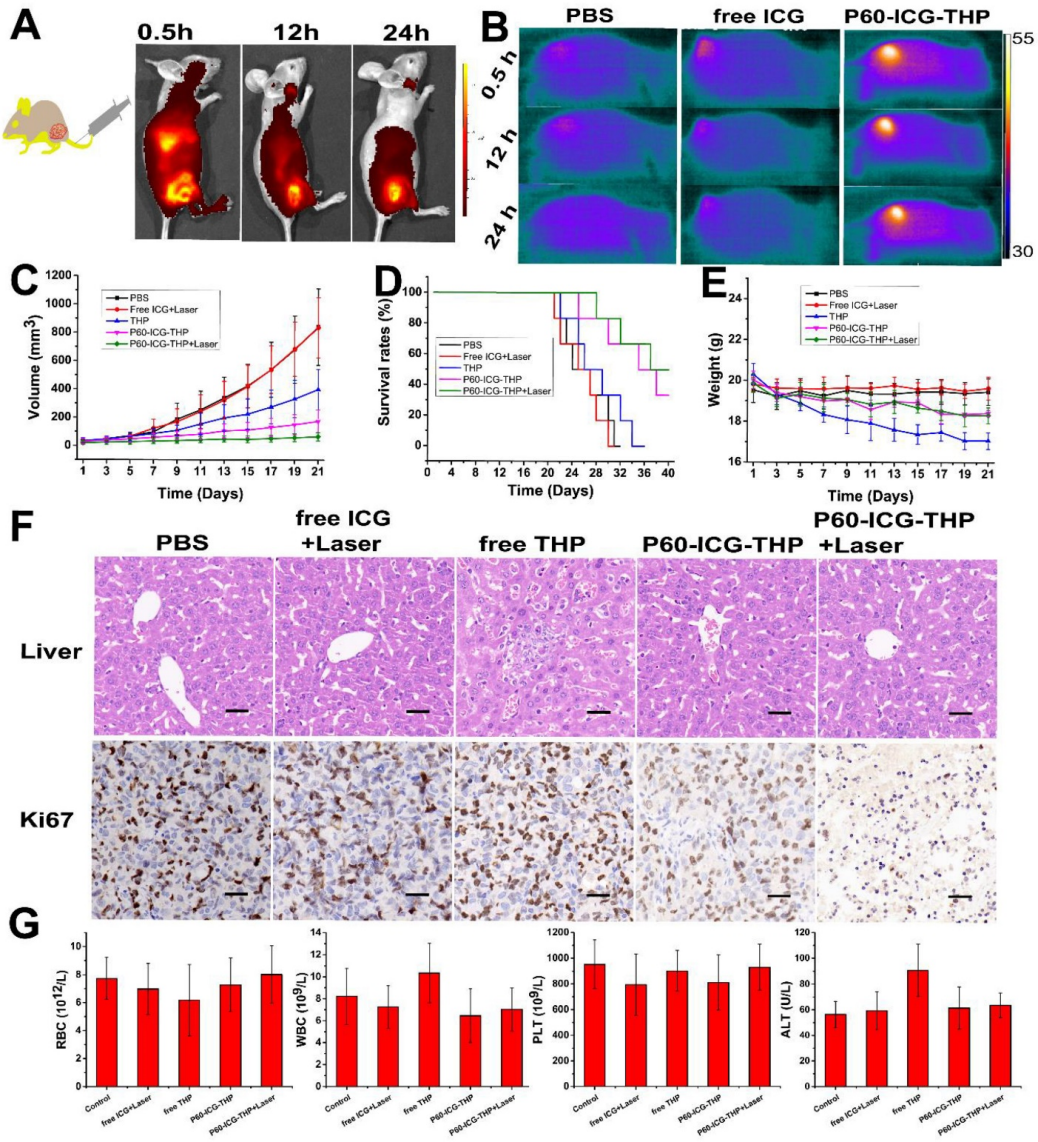
** (A)** *In vivo* imaging and biodistribution in nude mice bearing MDA-MB-231 tumors after tail vein injection of P60-ICG-THP. **(B)** IR thermal images of nude mice bearing MDA-MB-231 tumors after tail vein injection of PBS, ICG, and P60-ICG-THP and exposed to an 808-nm laser for 5 min. **(C)** Tumor growth curves of different groups after different treatments.** (D)** Cumulative survival rates of mice bearing MDA-MB-231 tumors after different treatments.** (E)** Body weight changes in the different groups of mice during different treatments. **(F)** HE staining of liver collected from different groups. The red arrows show hepatocellular injury. The bottom row shows Ki67 staining of tumor sections collected from different treatment groups. Scale bar = 60 μm. **(G)** Blood biochemical levels and hematological parameters of the mice after treatment. (n = 3, *p < 0.01).

ICG is often used as an organ function test agent such as for blood volume determination, cardiac output, and hepatic function; however, it is rapidly excreted from the body via the liver and kidney and is very difficult to achieve its accumulation in the diseased region 48-49. After mice bearing MDA-MB-231 tumors were injected with P60-ICG-THP, fluorescence live images were acquired at different time points (Figure 7A). The emission intensity of THP was found to be enriched in tumors, illustrating that P60-ICG-THP could accomplish the tumor-targeted delivery of anticancer agents through the enhanced permeability and retention (EPR) effect. Recently, a study has reported that nanoparticle transport through gaps between endothelial cells in the tumor blood vessel was found to be up to 2,000 nm 50. Infrared thermal imaging was performed on nude mice bearing MDA-MB-231 tumors exposed to an 808-nm laser for 5 min after tail vein injections of PBS, ICG, and P60-ICG-THP to detect the amount of enriched ICG (Figure 7B). The temperature of the tumor treated with P60-ICG-THP was the highest and reached 54.5°C, which was sufficiently high to ablate malignant cells. In the PBS and free ICG groups, the temperature reached 38.5°C and 39.6°C, respectively. These results fully illustrated that free ICG was eliminated from the body of the mice within a short time. With the help of our delivery system, ICG could accumulate in the tumors for prolonged duration.

### Chemo-photothermal-starvation combination therapy for highly efficient anticancer effect *in vivo*

MDA-MB-231 tumor-bearing nude mice were divided into five groups (PBS, free ICG+Laser, free THP, P60-ICG-THP, and P60-ICG-THP+Laser). PBS, free ICG, free THP, and P60-ICG-THP were injected into the mice via tail vein injection every alternate day, and a 5-min laser irradiation was carried out 12 h after the injection of free ICG or P60-ICG-THP (Figure S12). The tumor growth curves are shown in Figure 7C. The tumor volume of mice treated with PBS or free ICG plus laser irradiation increased sharply within 21 days, with no statistical difference (p > 0.05), suggesting that ICG and laser irradiation were safe. In contrast, tumor growth was inhibited in the other groups. Not surprisingly, the antitumor effect of P60-ICG-THP+laser, which combined photothermal therapy, chemotherapy, and starvation cancer therapy, was the best, and the results were consistent with those of the *in vitro* antitumor experiments. The median survival time of the mice treated with PBS was 26 days, and treatment with free ICG plus laser irradiation or free THP induced no significant increase in the survival rate (Figure 7D). P60-ICG-THP and P60-ICG-THP+laser treatment increased the survival rate to 37 and 38 days, respectively. Ki67, a nuclear protein associated with cellular proliferation, was detected by immunohistochemistry (second row of Figure 7F). Most tumor cells were stained brown in the PBS, free ICG+Laser, and free THP groups, whereas the number of brown cells was significantly reduced in the P60-ICG-THP and P60-ICG-THP+laser groups. In particular, only a few brown tumor cells were observed after treatment with P60-ICG-THP+laser, illustrating that the active proliferation of tumor cells was inhibited. Therefore, we conclude that P60-ICG-THP showed a significantly enhanced antitumor effect through chemo-photothermal-starvation combination therapy.

Finally, the body weight, blood biochemical levels, hematological parameters, and hematoxylin/eosin (HE) staining of five visceral organs from tumor-bearing nude mice were evaluated to determine the safety of P60-ICG-THP *in vivo*. Free THP caused significant body weight loss, while the other treatments induced no significant change compared with that in the control group (Figure 7E). As shown in Figure 7G, the level of alanine aminotransferase (ALT) was significantly increased in mice treated with free THP compared with that in the other groups. No evident differences in the other hematological parameters, such as red blood cells (RBC), white blood cells (WBC), and platelets (PLT), were observed. HE staining of five visceral organs is shown in the first row of Figure 7F and Figure S14. Only liver cell injury was observed in the free THP group, while injury was not observed in the other tissues. Thus, we confirmed that P60-ICG-THP possessed high biocompatibility and was minimally toxic or non-toxic to normal tissues and organs.

## Conclusion

In summary, we screened two antitumor drugs that could sequentially trigger self-assembling peptides (P60) to form a large (500-1000 nm), negatively charged drug delivery system (P60-ICG-THP). When P60-ICG-THP localizes in the tumor, its low pH could rapidly trigger the size/charge switches of P60-ICG-THP to achieve the precise subcellular delivery of the multiple loaded drugs. THP is then delivered to the cell nuclei, while ICG is mainly enriched in the blood vessels, tissue spaces, and cytoplasm of the tumors. Furthermore, the non-homogeneous particles accumulating in tumor blood vessels can selectively block the supply of nutrients and oxygen and kill cancer cells. Therefore, our drug delivery system can provide a new strategy to ingeniously combine chemotherapy, photothermal therapy, and cancer starvation therapy for the development of highly efficient antitumor agents.

## Supplementary Material

Supplementary figures and tables.

## References

[1] Al-Ahmady ZS, Jasim D, Ahmad SS, Wong R, Haley M, Coutts G (2019). Selective Liposomal Transport through Blood Brain Barrier Disruption in Ischemic Stroke Reveals Two Distinct Therapeutic Opportunities. ACS Nano.

[2] Jin Z, Nguyen KT, Go G, Kang B, Min HK, Kim SJ (2019). Multifunctional Nanorobot System for Active Therapeutic Delivery and Synergistic Chemo-photothermal Therapy. Nano Lett.

[3] Jiang X, Wang G, Liu R, Wang Y, Wang Y, Qiu X (2013). RNase non-sensitive and endocytosis independent siRNA delivery system: delivery of siRNA into tumor cells and high efficiency induction of apoptosis. Nanoscale.

[4] Ramasamy T, Ruttala HB, Kahraj K, Poudel K, Jin SG, Choi HG (2019). Polypeptide Derivative of Metformin with the Combined Advantage of a Gene Carrier and Anticancer Activity. ACS Biomater Sci Eng.

[5] Jiang X, Fan X, Xu W, Zhao C, Wu H, Zhang R (2019). Self-assembled peptide nanoparticles responsive to multiple tumor microenvironment triggers provide highly efficient targeted delivery and release of antitumor drug. J Control Release.

[6] Davoodi P, Lee LY, Xu Q, Sunil V, Sun Y, Soh S (2018). Drug delivery systems for programmed and on-demand release. Adv Drug Deliv Rev.

[7] Chen D, Zhang G, Li R, Guan M, Wang X, Zou T (2018). Biodegradable, Hydrogen Peroxide, and Glutathione Dual Responsive Nanoparticles for Potential Programmable Paclitaxel Release. J Am Chem Soc.

[8] Mi P (2020). Stimuli-responsive nanocarriers for drug delivery, tumor imaging, therapy and theranostics. Theranostics.

[9] Wang X, Wang X, Jin S, Muhammad N, Guo Z (2019). Stimuli-Responsive Therapeutic Metallodrugs. Chem Rev.

[10] Aibani N, Nesbitt H, Marino N, Jurek J, O'Neill C, Martin C (2018). Electroneutral polymersomes for combined cancer chemotherapy. Acta Biomater.

[11] Ji C, Gao Q, Dong X, Yin W, Gu Z, Gan Z (2018). A Size-Reducible Nanodrug with an Aggregation-Enhanced Photodynamic Effect for Deep Chemo-Photodynamic Therapy. Angew Chem Int Ed Engl.

[12] Song Q, Yin Y, Shang L, Wu T, Zhang D, Kong M (2017). Tumor Microenvironment Responsive Nanogel for the Combinatorial Antitumor Effect of Chemotherapy and Immunotherapy. Nano Lett.

[13] Dai W, Wang X, Song G, Liu T, He B, Zhang H (2017). Combination antitumor therapy with targeted dual-nanomedicines. Adv Drug Deliv Rev.

[14] Rees P, Wills JW, Brown MR, Barnes CM, Summers HD (2019). The origin of heterogeneous nanoparticle uptake by cells. Nat Commun.

[15] Chen G, Roy I, Yang C, Prasad PN (2016). Nanochemistry and Nanomedicine for Nanoparticle-based Diagnostics and Therapy. Chem Rev.

[16] Mosquera J, Garcia I, Liz-Marzan LM (2018). Cellular Uptake of Nanoparticles versus Small Molecules: A Matter of Size. Acc Chem Res.

[17] Donahue ND, Acar H, Wilhelm S (2019). Concepts of nanoparticle cellular uptake, intracellular trafficking, and kinetics in nanomedicine. Adv Drug Deliv Rev.

[18] Behzadi S, Serpooshan V, Tao W, Hamaly MA, Alkawareek MY, Dreaden EC (2017). Cellular uptake of nanoparticles: journey inside the cell. Chem Soc Rev.

[19] Mandl HK, Quijano E, Suh HW, Sparago E, Oeck S, Grun M (2019). Optimizing biodegradable nanoparticle size for tissue-specific delivery. J Control Release.

[20] Lu F, Wu SH, Hung Y, Mou CY (2009). Size effect on cell uptake in well-suspended, uniform mesoporous silica nanoparticles. Small.

[21] Jo DH, Kim JH, Lee TG, Kim JH (2015). Size, surface charge, and shape determine therapeutic effects of nanoparticles on brain and retinal diseases. Nanomedicine.

[22] Vigderman L, Manna P, Zubarev ER (2012). Quantitative replacement of cetyl trimethylammonium bromide by cationic thiol ligands on the surface of gold nanorods and their extremely large uptake by cancer cells. Angew Chem Int Ed Engl.

[23] Zhang M, Chen X, Li C, Shen X (2019). Charge-reversal nanocarriers: An emerging paradigm for smart cancer nanomedicine. J Control Release.

[24] Duan X, Li Y (2013). Physicochemical characteristics of nanoparticles affect circulation, biodistribution, cellular internalization, and trafficking. Small.

[25] Xiao K, Li Y, Luo J, Lee JS, Xiao W, Gonik AM (2011). The effect of surface charge on *in vivo* biodistribution of PEG-oligocholic acid based micellar nanoparticles. Biomaterials.

[26] Habibi N, Kamaly N, Memic A, Shafiee H (2016). Self-assembled peptide-based nanostructures: Smart nanomaterials toward targeted drug delivery. Nano Today.

[27] Kim J, Narayana A, Patel S, Sahay G (2019). Advances in intracellular delivery through supramolecular self-assembly of oligonucleotides and peptides. Theranostics.

[28] Booth R, Insua I, Bhak G, Montenegro J (2019). Self-assembled micro-fibres by oxime connection of linear peptide amphiphiles. Org Biomol Chem.

[29] Jin H, Wan C, Zou Z, Zhao G, Zhang L, Geng Y (2018). Tumor Ablation and Therapeutic Immunity Induction by an Injectable Peptide Hydrogel. ACS Nano.

[30] Qi GB, Gao YJ, Wang L, Wang H (2018). Self-Assembled Peptide-Based Nanomaterials for Biomedical Imaging and Therapy. Adv Mater.

[31] Wei G, Su Z, Reynolds NP, Arosio P, Hamley IW, Gazit E (2017). Self-assembling peptide and protein amyloids: from structure to tailored function in nanotechnology. Chem Soc Rev.

[32] Wei YS, Liao RF, Mahmood AA, Xu HB, Zhou QB (2017). pH-responsive pHLIP (pH low insertion peptide) nanoclusters of superparamagnetic iron oxide nanoparticles as a tumor-selective MRI contrast agent. Acta Biomater.

[33] Falcone N, Kraatz HB (2018). Supramolecular Assembly of Peptide and Metallopeptide Gelators and Their Stimuli-Responsive Properties in Biomedical Applications. Chemistry.

[34] Roth-Konforti ME, Comune M, Halperin-Sternfeld M, Grigoriants I, Shabat D, Adler-Abramovich L (2018). UV Light-Responsive Peptide-Based Supramolecular Hydrogel for Controlled Drug Delivery. Macromol Rapid Commun.

[35] Aszodi A, Hauser N, Studer D, Paulsson M, Hiripi L, Bosze Z (1996). Cloning, sequencing and expression analysis of mouse cartilage matrix protein cDNA. Eur J Biochem.

[36] Dames SA, Kammerer RA, Wiltscheck R, Engel J, Alexandrescu AT (1998). NMR structure of a parallel homotrimeric coiled coil. Nat Struct Biol.

[37] Ha BH, Boggon TJ (2018). The crystal structure of pseudokinase PEAK1 (Sugen kinase 269) reveals an unusual catalytic cleft and a novel mode of kinase fold dimerization. J Biol Chem.

[38] Fan X, Zhao F, Wang X, Wu G (2016). Doxorubicin-triggered self-assembly of native amphiphilic peptides into spherical nanoparticles. Oncotarget.

[39] Kato Y, Ozawa S, Miyamoto C, Maehata Y, Suzuki A, Maeda T (2013). Acidic extracellular microenvironment and cancer. Cancer Cell Int.

[40] Gatenby RA, Gillies RJ (2004). Why do cancers have high aerobic glycolysis?. Nat Rev Cancer.

[41] Jia L, Pang M, Fan M, Tan X, Wang Y, Huang M (2020). A pH-responsive Pickering Nanoemulsion for specified spatial delivery of Immune Checkpoint Inhibitor and Chemotherapy agent to Tumors. Theranostics.

[42] Yu Z, Zhou P, Pan W, Li N, Tang B (2018). A biomimetic nanoreactor for synergistic chemiexcited photodynamic therapy and starvation therapy against tumor metastasis. Nat Commun.

[43] Gao P, Shi M, Wei R, Pan W, Liu X, Li N (2020). A biomimetic MOF nanoreactor enables synergistic suppression of intracellular defense systems for augmented tumor ablation. Chem Commun (Camb).

[44] Yu SJ, Chen ZW, Zeng X, Chen XS, Gu Z (2019). Advances in nanomedicine for cancer starvation therapy. Theranostics.

[45] Li SP, Jiang Q, Liu SL, Zhang YL, Tian YH, Song C (2018). A DNA nanorobot functions as a cancer therapeutic in response to a molecular trigger *in vivo*. Nat Biotechnol.

[46] Zhang C, Ni DL, Liu YY, Yao HL, Bu WB, Shi JL (2019). Magnesium silicide nanoparticles as a deoxygenation agent for cancer starvation therapy (vol 12, pg 378, 2017). Nat Nanotechnol.

[47] Zou M, Xu P, Wang L, Wang L, Li T, Liu C (2020). Design and construction of a magnetic targeting pro-coagulant protein for embolic therapy of solid tumors. Artif Cells Nanomed Biotechnol.

[48] Yang J, Tao HS, Cai W, Zhu W, Zhao D, Hu HY (2018). Accuracy of actual resected liver volume in anatomical liver resections guided by 3-dimensional parenchymal staining using fusion indocyanine green fluorescence imaging. J Surg Oncol.

[49] Yamada Y, Ohno M, Fujino A, Kanamori Y, Irie R, Yoshioka T (2019). Fluorescence-Guided Surgery for Hepatoblastoma with Indocyanine Green. Cancers (Basel).

[50] Sindhwani S, Syed AM, Ngai J, Kingston BR, Maiorino L, Rothschild J (2020). The entry of nanoparticles into solid tumours. Nat Mater.

