# Supplementary material for: In situ tumor-triggered subcellular precise delivery of multi-drugs for enhanced chemo-photothermal-starvation combination antitumor therapy
Source: Theranostics. 2020 Oct 27;10(26):12158–73. doi: 10.7150/thno.52000 (PMC7667678; doi:10.7150/thno.52000)
Supplement: Supplementary file 1 — Supplementary figures and tables. [file thnov10p12158s1.pdf]

Electronic Supplementary Information

In situ tumor-triggered subcellular precise delivery of multi-drugs for enhanced chemo-photothermal-starvation combination antitumor therapy

*Xinglu Jiang[1], Xiaobo Fan[1], Rui Zhang[1], Wei Xu[1], Hailu Wu[1], Fengfeng Zhao[2], Han Xiao[1], Chen Zhang[2], Chenggui Zhao[2], Guoqiu Wu[2,3,]**

1. Medical School of Southeast University, Nanjing 210009, People's Republic of China.
2. Center of Clinical Laboratory Medicine, Zhongda Hospital, Southeast University, Nanjing 210009, People's Republic of China.
3. Jiangsu Provincial Key Laboratory of Critical Care Medicine, Southeast University, Nanjing 210009, People's Republic of China.

*Corresponding author. E-mail address: nationball@163.com (G. Wu).

Keywords: subcellular precise delivery, particle size/surface charge switches, tumor pH triggered, tumor combined therapy, self-assembling peptide

Supporting Figures and Tables

Table S1. The physicochemical property of peptides

Abbr.	Origin from	Molecular Weigh	Isoelectric Point	Net charge in pH=7	Hydrophilia
P26	Homo Peak- 1	2886.5 g/mol	9.7	2.0	0.1
P34	Homo matrilin-1	3797.5 g/mol	10.2	2.0	0.2
P60	Homo matrilin-1/ Peak- 1	6666 g/mol	10.2	4.1	0.2

Table S2. The water soluble antitumor drug

	No aryl	< C10	C10-C20	C20-C30	C30-C50
HCl or H_2SO_4	Mechlorethamine hydrochloride	Nimustine hydrochloride	Bendamustine hydrochloride	Doxorubicin hydrochloride	Aclarubicin Hydrochloride
Na^+		Saccharin sodium dihydrate	Combretastatin A4 disodium phosphate	Pemetrexed disodium	Indocyanine green
Hydrophilic group	Busulfan	Floxuridine	Fludarabine phosphate	Colchicine	

Table S3. The water insoluble antitumor drug

Categories	No aryl	< C10	C10-C20	C20-C30	C30-C50
Hormonal			Letrozole	Exemestane	Fulvestrant
Antibiotics				Adriamycin	Pirarubicin
Alkylating	Carmustine	Dacarbazine		Mizolastine	
Antimetabolites		6-Thioguanine	Clofarabine	Methotrexate	
Natural			Cantharidin	7-Ethyl-10-hydroxycamptothecin	Paclitaxel
Others	Cisplatin		Lenalidomide	Vemurafenib	

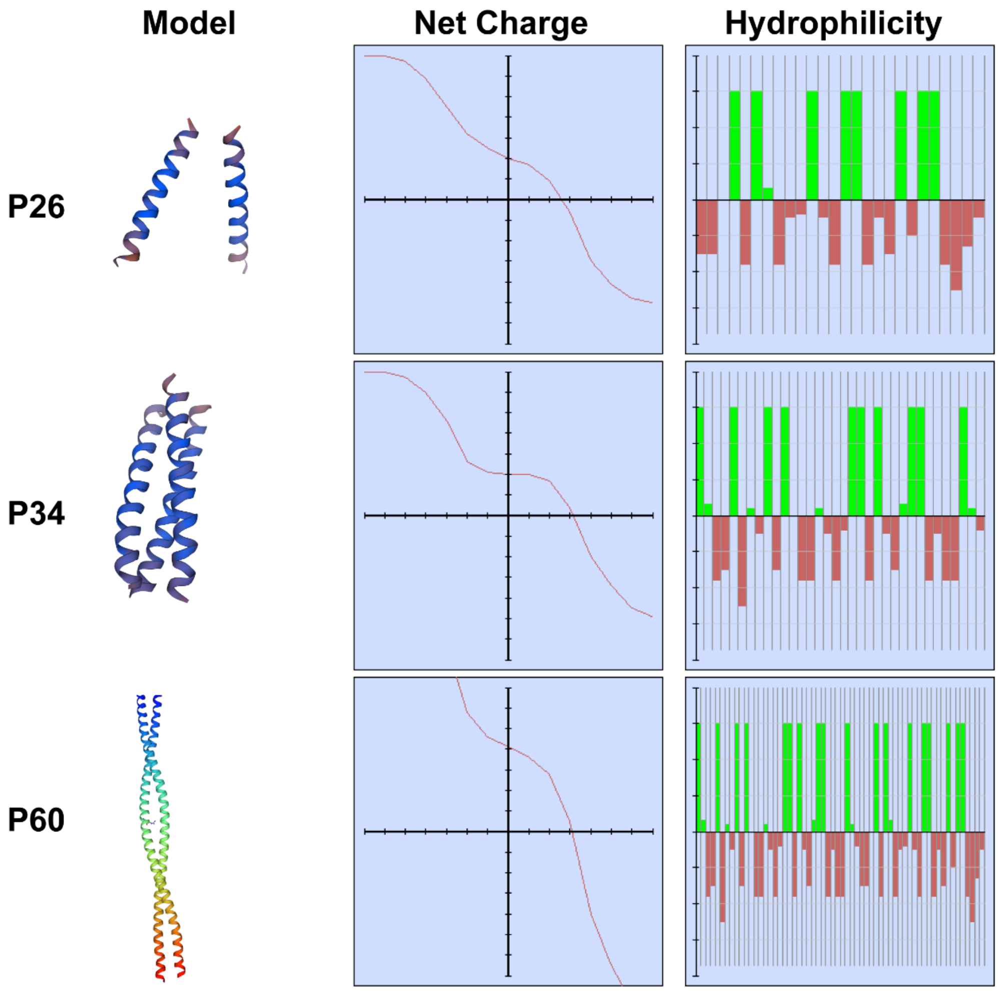

Figure S1. The three dimensional structure, net charge and hydrophilicity of peptides.

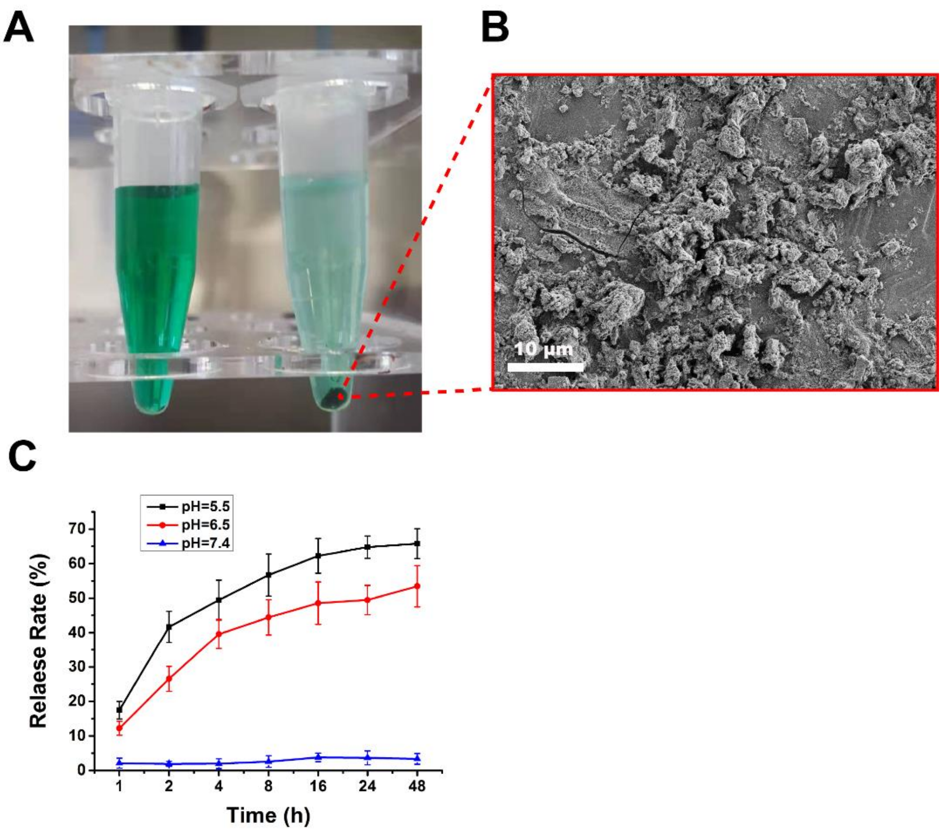

Figure S2. A) The light photograph of ICG and P60-ICG after centrifuge. B) SEM image of P60-ICG. 3) in vitro release kinetics of pirarubicin from P60-ICG-THP in the different pH.

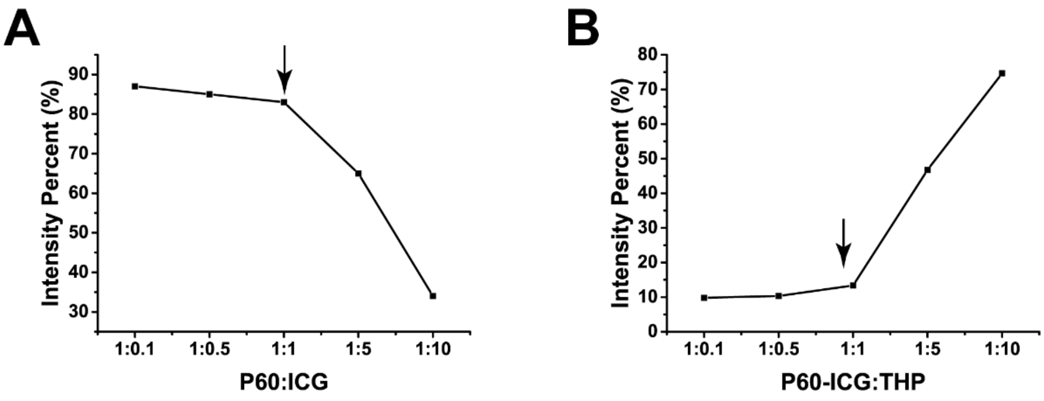

Figure S3. A) The emission intensity percent of ICG before and after assembled by P60 in different mass ratio between P60 and ICG. B) The emission intensity percent of THP before and after assembled by P60-ICG

in different mass ratio between P60-ICG and THP.

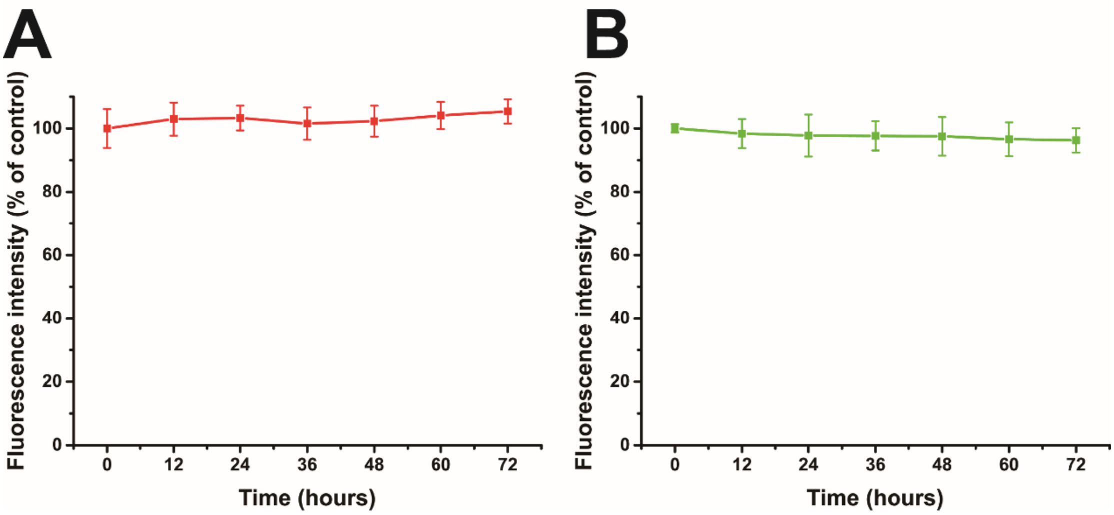

Figure S4. The fluorescence intensity of ICG and THP of our delivery system (P6-ICG-THP) was measured in the different time to assess its stable in serum.

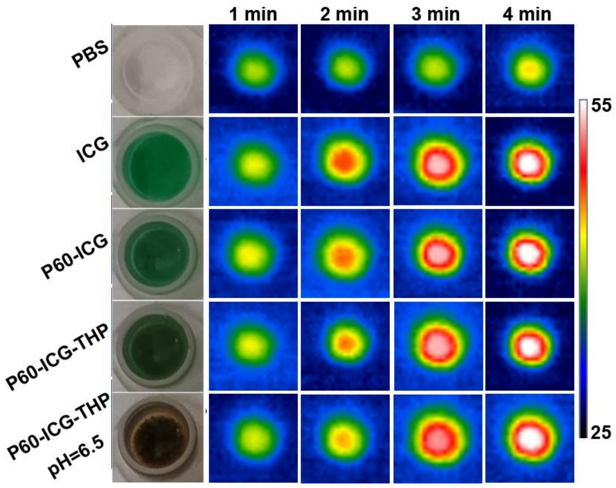

Figure S5. Left: light photograph of PBS, ICG, P60-ICG, P60-ICG-THP and P60-ICG-THP at pH=6.5. Right: the infrared thermographic maps of PBS, ICG, P60-ICG, P60-ICG-THP and P60-ICG-THP at pH=6.5 under

continuous 808 nm laser irradiation at a power intensity of 1 W/cm2 for 1, 2, 3, 4 and 5 min.

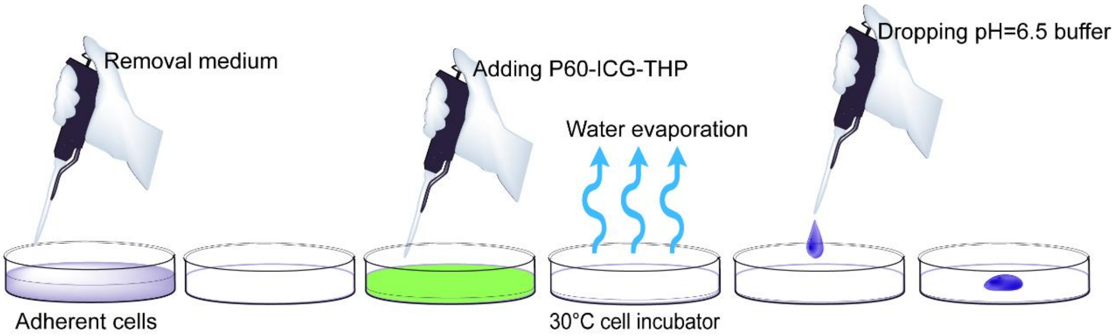

Figure S6. The flow diagram of MDA-MB-231 incubated with P60-ICG-THP Microrobots after a drop of pH=6.5 buffer triggered in the same culture dish.

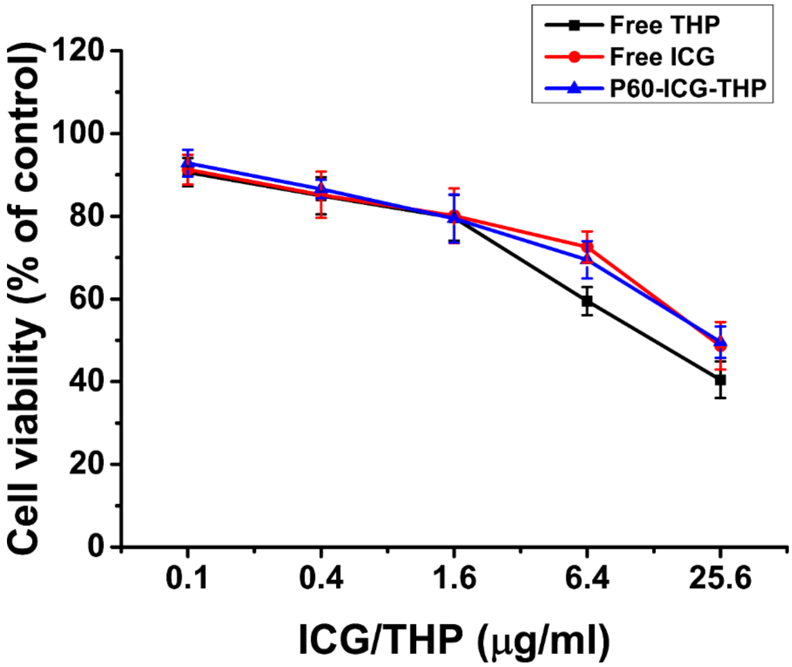

Figure S7. Cell viability of MDA-MB-231 incubated with different concentrations of free THP, free ICG, and P60-ICG-THP at pH 7.4 with laser.

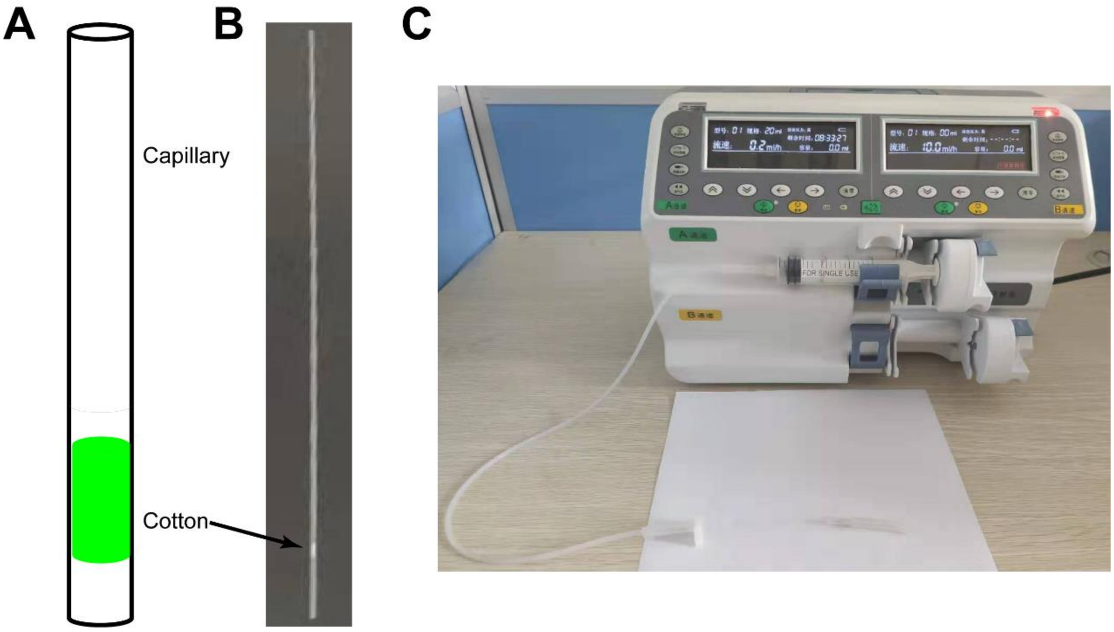

Figure S8. The equipment of starving therapy in vitro, such as capillary tube and micro-injection pump.

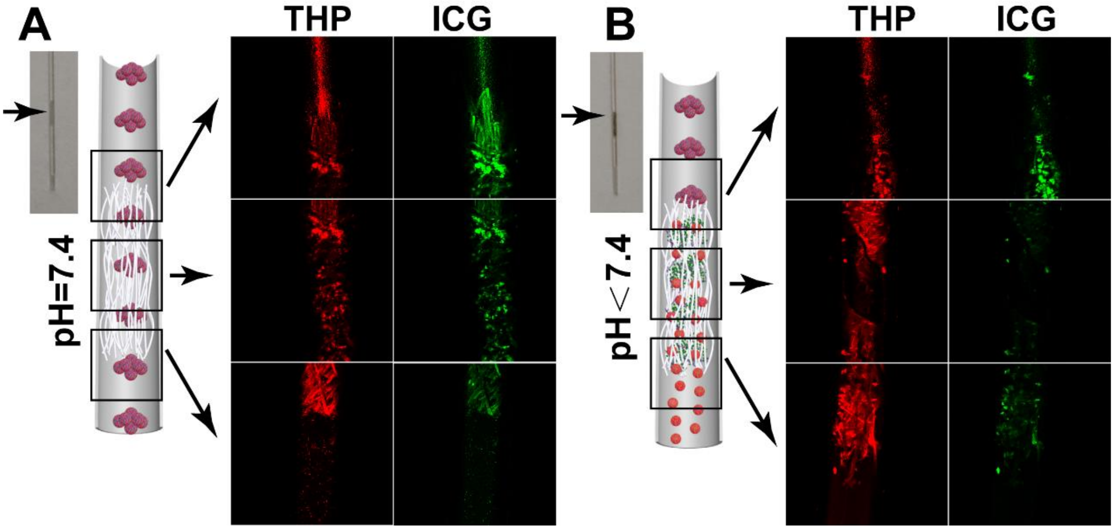

Figure S9. The model of the starvation therapy in vitro. Inset: photographs of capillary tube at (A) pH=7.4 or (B) pH<7.4; Middle: schematic illustration of starvation therapy in vitro; Right: confocal

fluorescence images to trace ICG (green) and THP (red) in the capillary tube after P60-ICG-THP flowing past.

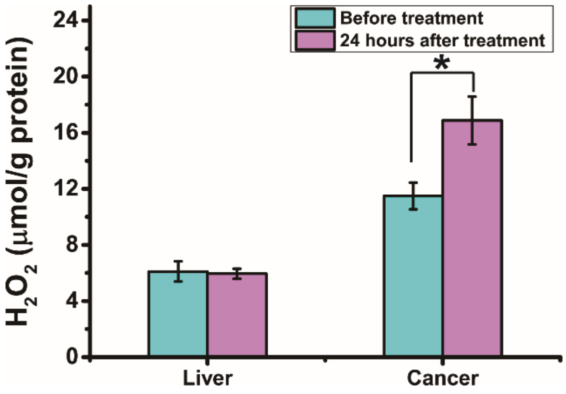

Figure S10. H_2O_2 in the liver and tumor tissues from xenograft models treated with P60-ICG-THP (n=3, *p<0.01)

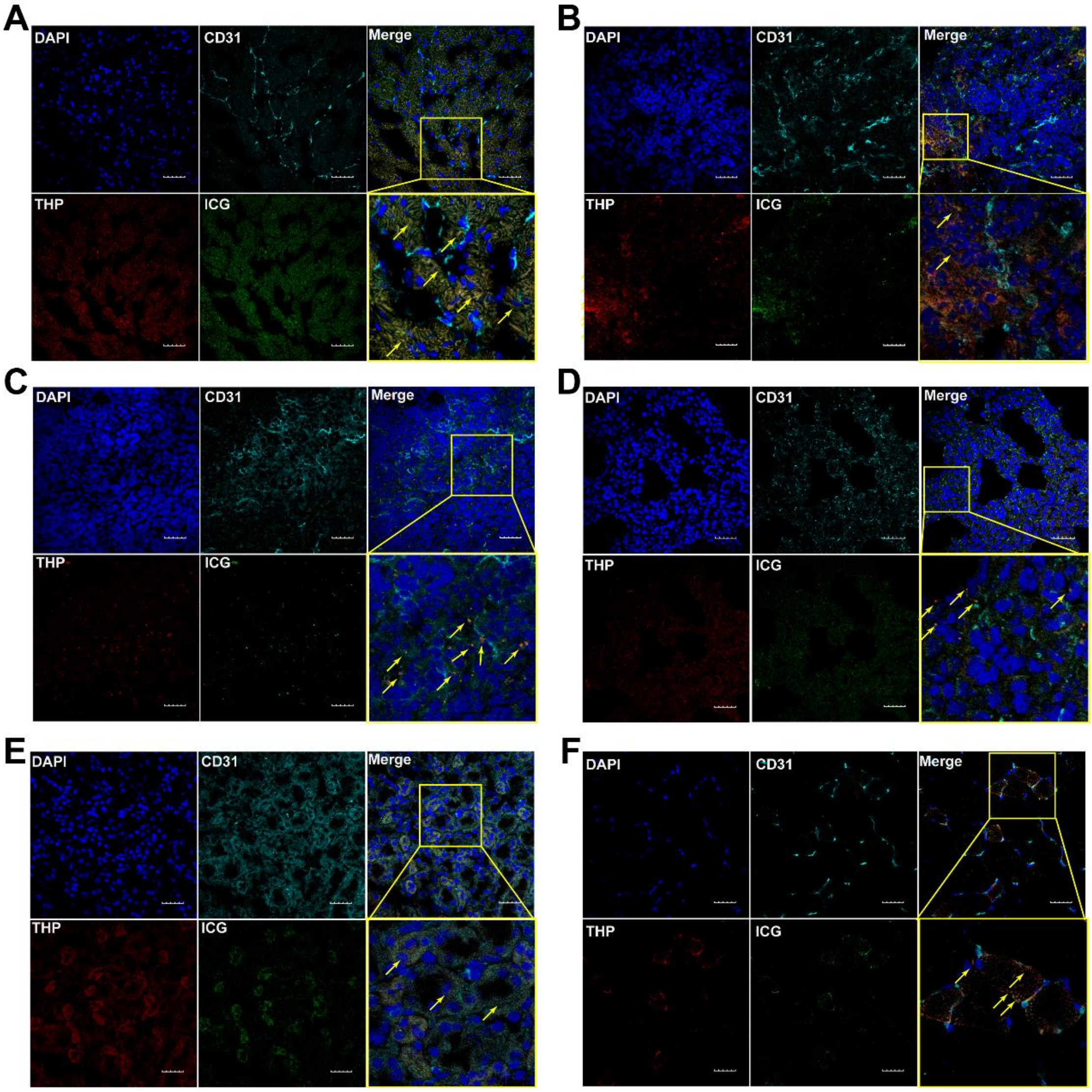

Figure S11. Confocal fluorescence images of heart (A), liver (B), spleen (C), lung (D), kidney (E) and muscle (F) from xenograft model treated with P60-ICG-THP. Cells stained with DAPI and vascular endothelial cell stained with FITC-CD31; DAPI (blue), FITC (cyan), THP (red) and ICG (green) excited by 405 nm, 488 nm, 561and 633 nm, scale bar= 20 μm.

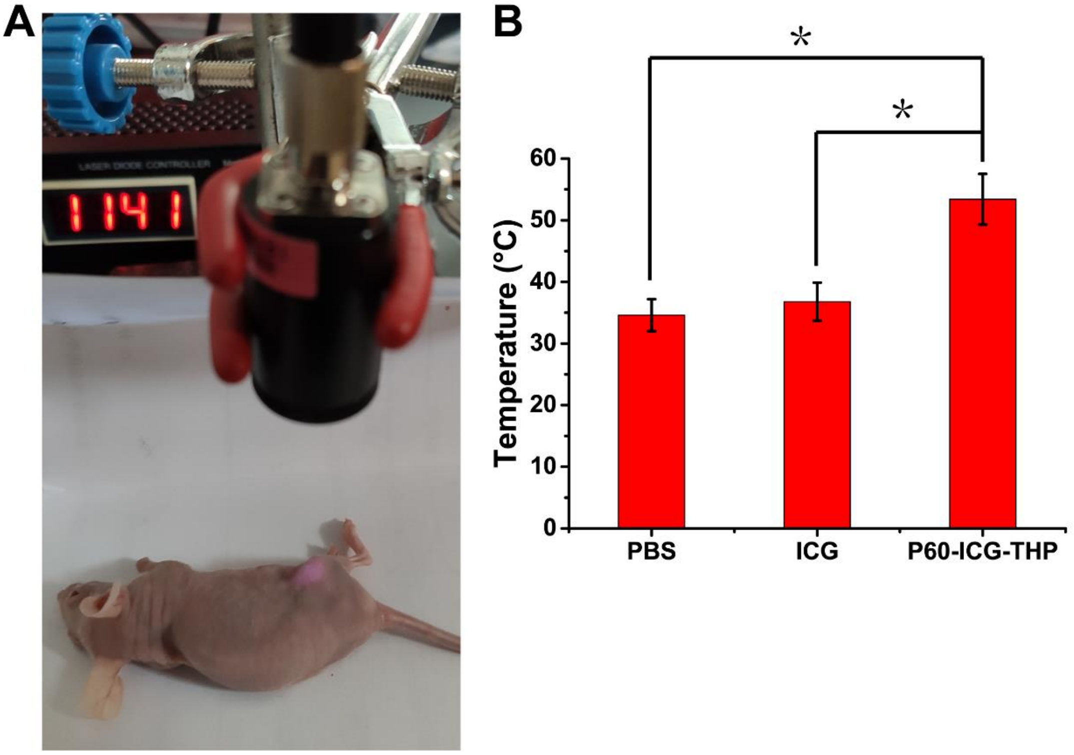

Figure S12. A) The light photograph of nude mice bearing MDA-MB-231 tumors after tail vein injection of PBS, ICG and P60-ICG-THP Microrobots and exposed to 808 nm laser. B) Maximum temperature profiles of nude mice bearing MDA-MB-231 tumors after tail vein injection of PBS, ICG and P60-ICG-THP Microrobots under continuous 808 nm laser irradiation at a power intensity of 1 W/cm2 for 5 min.

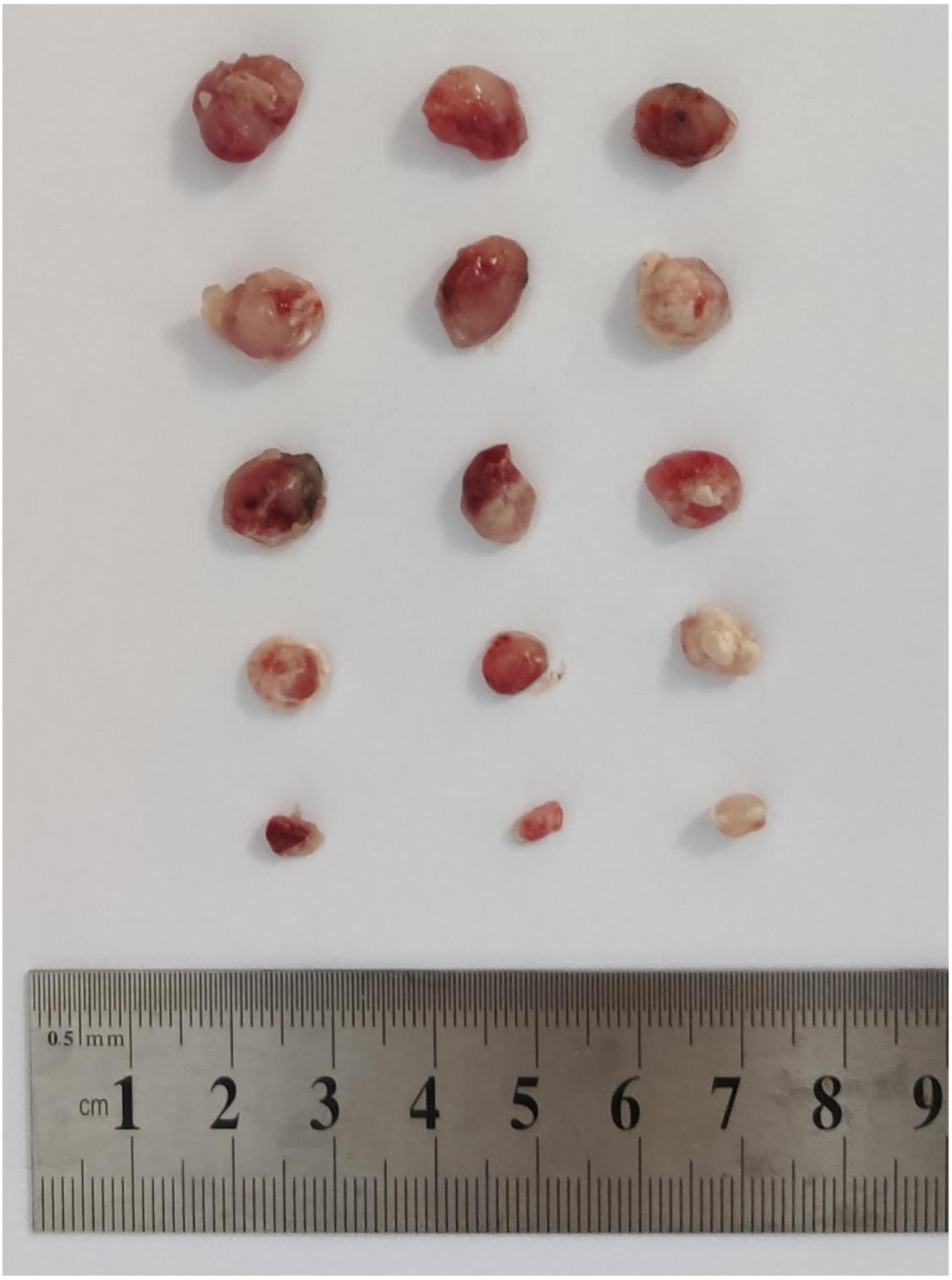

Figure S13. Representative pictures of the tumor blocks collected from nude mice bearing MDA-MB-231 tumors treated with PBS, free ICG+Laser, THP, P60-ICG-THP Microrobots and P60-ICG-THP Microrobots+Laser.

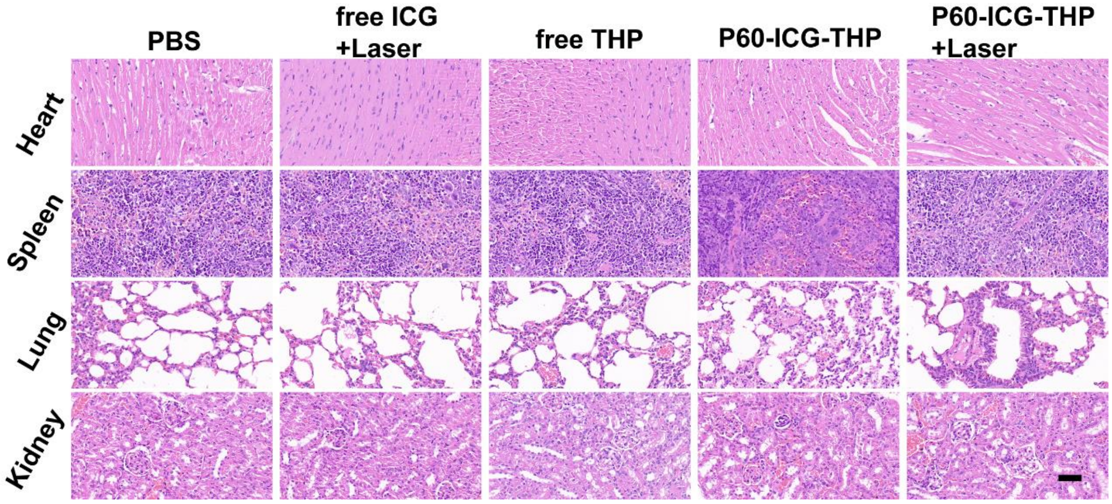

Figure S14. HE staining of heart, spleen, lung and kidney collected from nude mice bearing MDA-MB-231 tumors treated with PBS, free ICG+Laser, THP, P60-ICG-THP Microrobots and P60-ICG-THP Microrobots+Laser.
